# Applying Gaussian Process Machine Learning and Modern Probabilistic Programming to Satellite Data to Infer CO_2_ Emissions

**DOI:** 10.1021/acs.est.4c09395

**Published:** 2025-02-24

**Authors:** Seongeun Jeong, Sofia D. Hamilton, Matthew S. Johnson, Dien Wu, Alexander J. Turner, Marc L. Fischer

**Affiliations:** † Energy Analysis and Environmental Impacts Division, Lawrence Berkeley National Laboratory, Berkeley, California 94720, United States; ‡ Earth Science Division, NASA Ames Research Center, Moffett Field, California 94035, United States; § Division of Geological and Planetary Sciences, California Institute of Technology, Pasadena, California 91125, United States; ∥ Department of Atmospheric and Climate Science, University of Washington, Seattle, Washington 98195, United States

**Keywords:** carbon dioxide, satellite, emissions, machine learning, Gaussian process, probabilistic programming

## Abstract

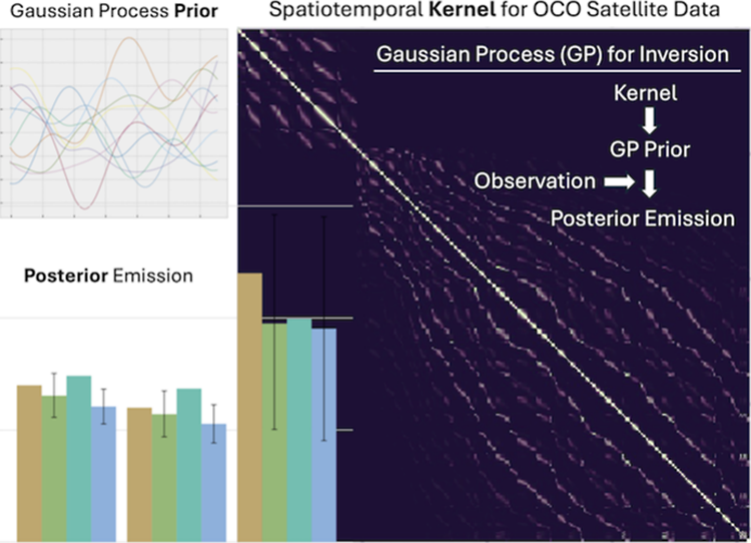

Satellite data provides essential insights into the spatiotemporal distribution of CO_2_ concentrations. However, many atmospheric inverse models fail to adequately incorporate the spatial and temporal correlations inherent in satellite observations and often lack rigorous methods for estimating parameters like spatial length scales. We introduce an inference model that processes the spatiotemporal covariance in satellite data and estimates hyperparameters such as covariance length scales. Our approach uses the Gaussian process (GP) machine learning (ML) and modern probabilistic programming languages (PPLs) to perform atmospheric inversions of emissions from satellite data. We develop a GP ML inversion system based on modern PPLs and the GEOS-Chem chemical transport model, simulating atmospheric CO_2_ concentrations corresponding to the Orbiting Carbon Observatory-2/3 (OCO-2/3) data for July 2020. In our supervised learning framework, we treat the GEOS-Chem simulated data set as the target, with predictors derived by scaling the target with sector-specific factors hidden from the GP machine. Our results show that the GP model, combined with GPU-enabled PPLs, effectively retrieves true emission scaling factors and infers noise levels concealed within the data. This suggests that our method could be applied over larger areas with more complex covariance structures, enabling comprehensive analysis of the spatiotemporal patterns observed in OCO-2/3 and similar satellite data sets.

## Abstract 2

This work demonstrates a new approach to atmospheric inverse modeling of CO_2_ emissions using Gaussian Process machine learning and modern probabilistic programming languages.

## 1 Introduction

Accurate evaluation of CO_2_ emission inventories is crucial for meeting climate goals.^1^ Advancements in monitoring technologies and emission inference methodologies have made it increasingly feasible to track CO_2_ emissions with higher precision and resolution, addressing climate change challenges by providing observation-based emission estimates for policymakers to evaluate their official inventories. However, the current methods of tracking these emissions are often limited by spatial and temporal coverage, resulting in a critical gap in our understanding and management of their climate impact. While the bottom-up inventory approach for CO_2_ emissions can provide a granular understanding of emission sources, they have biases/errors and may miss sources, and these uncertainties in emission estimates increase with finer spatial scales.^2−4^ Ground-based observation strategies (e.g., tall tower observations) provide long-term monitoring of surface emissions but lack spatial coverage, limiting their ability to estimate emissions from large areas.

Satellites offer a distinct advantage over ground-based in situ measurement networks and aircraft campaigns by providing extensive spatiotemporal coverage of observations over large regions.^5,6^ This broad coverage can capture the varied emission profiles across different landscapes, from energy-intensive industrial areas to carbon-sequestering forests and agricultural lands. In combination with atmospheric inverse modeling techniques, satellite data can enhance CO_2_ monitoring by effectively resolving emission sources in space and time.^7^

**Figure 1:**
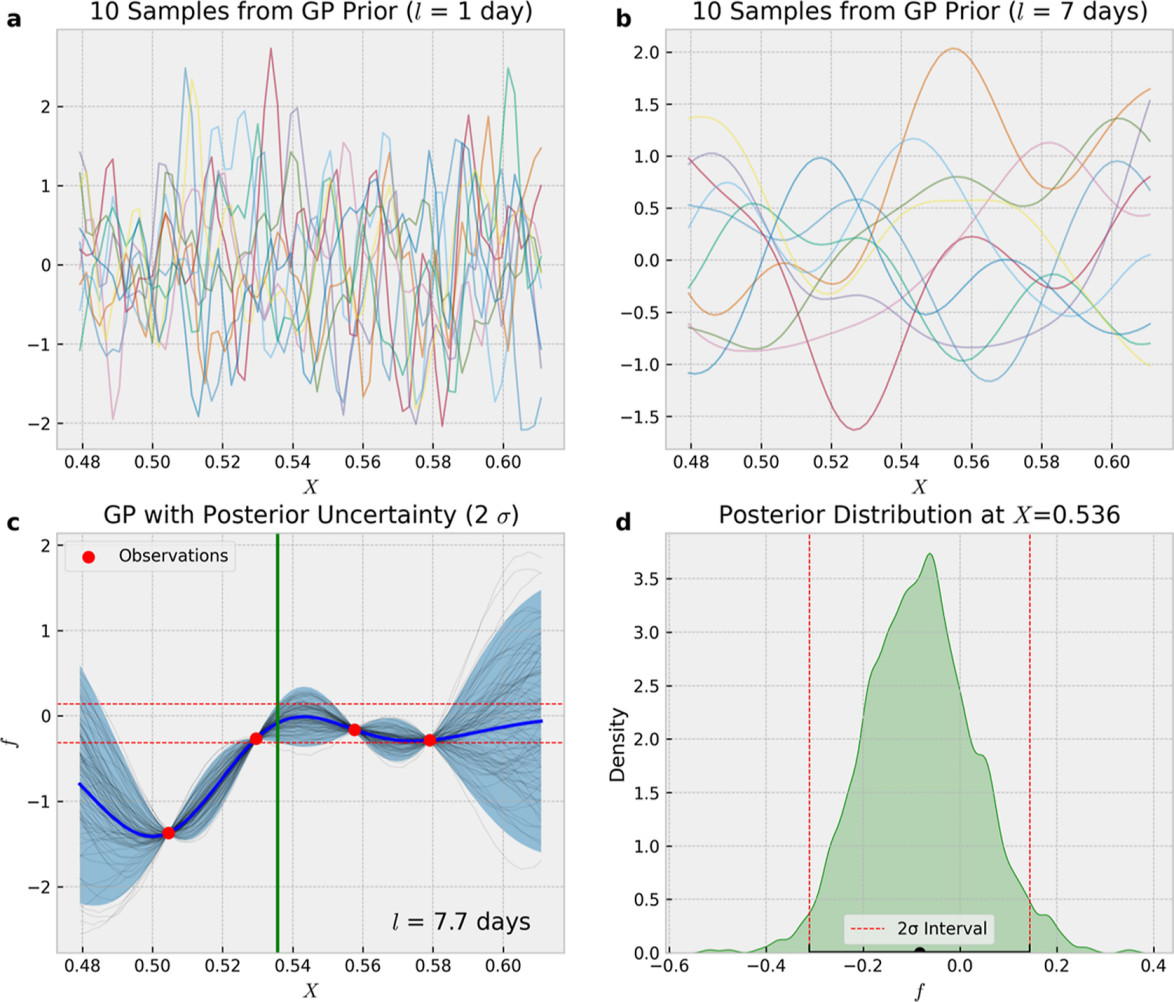
Example of sampling from a GP prior and GP posterior predictions: (a) 10 samples from a GP prior with a length scale (*l*) of 1 day, (b) samples from a GP prior with a length scale of 7 days, (c) the posterior distribution after incorporating four observations, and (d) the posterior distribution at X = 0.536, displaying uncertainty bounds corresponding to the light blue area in (c). The *X*-axis in panels (a–c) represents the normalized time, with 0.5 corresponding to the midpoint of the year 2020. The probability distribution corresponding to the vertical slice at *X* = 0.536 in (c) is depicted in (d). The two horizontal red dotted lines in (c) correspond to the red vertical lines in (d). In (c), we assume no noise, resulting in the posterior mean (blue line) precisely fitting the observations. In this example, we treated the GC model simulated CO_2_ background-subtracted concentrations as observations.

While satellite-based atmospheric inverse modeling provides an advanced method for quantifying CO_2_ emissions, using satellite observations in inversions introduces two principal challenges: (1) incorporating the spatiotemporal covariance structure inherent in satellite data, and (2) accurately estimating the hyperparameters, such as the length scale of this covariance and the observation noise associated with satellite data. Satellite observations contain both spatial and temporal characteristics that inform us about surface emissions. However, numerous inverse modeling studies have not fully incorporated both covariance structures.^4,8−11^ While some studies have accounted for both spatial and temporal covariances, they have not determined optimal hyperparameters that align with the observations.^12^ For example, the length scale parameter greatly influences the covariance, which in turn affects the estimation of the unknown functions we need to derive from the data, as illustrated in Figure 1.^13−16^ However, these parameters are often not estimated accurately (see Section 2.1.2).

We developed an atmospheric inversion system to fully utilize the spatiotemporal properties embedded in satellite data. Fully incorporating the spatiotemporal characteristics of satellite data involves understanding the hidden covariance structure. Our system infers this structure by estimating the covariance hyperparameters.^17^ This system is built based on the Gaussian process (GP) machine learning (ML) approach enabled by modern Probabilistic Programming Languages (PPLs). GP is an ML technique that treats predictions as probability distributions (illustrated in Figure 1; see Section 2.1), providing a measure of prediction uncertainty,^14,18,19^ which is ideal for atmospheric inverse modeling. Because a GP is a nonparametric model, it is not constrained by the linear assumptions adopted in most previous inverse studies.^20−23^ The assumption of linearity inherent in traditional inverse models may not always hold true when analyzing complex relationships between input data (such as model simulations) and satellite observations. Our GP-based approach is designed to overcome this limitation.^16,24^ GPs excel at capturing complex, nonlinear relationships between inputs and outputs without requiring predefined mathematical equations (see Section 2.1).^14^ They learn patterns directly from the data, with these underlying patterns and relationships represented through GP kernels (i.e., covariance structures), as detailed in Section 2.1.

PPLs have been used in previous studies,^10,23,25,26^ but more modern PPLs provide significantly improved capabilities to implement GP models. The two modern PPLs used in this study support graphics processing unit (GPU) computing and automatic differentiation tools (e.g., Google JAX) for high-performance computing (HPC) ML research (see Section 2 for details). This support for the HPC ML approach is critical because implementing a fully Bayesian GP model is computationally expensive.^15,27^

We demonstrate the developed atmospheric inversion system using a data set generated by the GEOS-Chem (GC) chemical transport model (CTM)^28,29^ corresponding to NASA’s orbiting carbon observatory (OCO) satellite observations. We illustrate how the kernels (i.e., covariance function) in GP models can be constructed to incorporate spatiotemporal covariance embedded in OCO-2/3 data. In particular, we show how the GP-based inversion system retrieves the true parameters, including the noise (similar to the model-data mismatch in the traditional inversion) hyperparameter, which was often prescribed in many previous studies.^11,30^

## 2 Data and Models

### 2.1 GP Machine Learning

#### 2.1.1 Background on GP

We implement atmospheric inverse modeling using a GP ML framework. GP is a flexible, nonparametric approach that directly defines prior probability distributions over functions.^13−15^ As in a typical atmospheric inversion, though applied to functions, this prior is updated to a posterior distribution using observed data. Within GPs, each point in the domain corresponds to a random variable. Collectively, these random variables define a joint Gaussian distribution. More details are provided using an example in Figure 1 below.

A GP is characterized by its mean *m*(**x**) and kernel *k*(**x**, **x**^′^)^14,15^
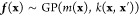
1
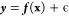
2where ***y*** is the target variable, which includes additive noise ϵ, where . In this work, ***y*** represents the noisy version of the GC CTM-simulated dry-air mole fraction time series. As demonstrated in eqs 1 and 2, the key difference between GPs and traditional linear inverse models, such as those in ref (23), is that in the GP model, the mean function is not derived from a predetermined linear form (typically model prediction based on prior emissions). Instead, it is an unknown function sampled from a distribution defined by a mean and a covariance. To aid understanding, consider that the function ***f***(**x**) has a mean of zero [i.e., *m*(**x**) = 0, a common assumption] and a covariance matrix **K**, which needs to be configured. The GP model exhibits a convenient yet powerful property: **K** is constructed using the kernel functions *k*(**x**, **x**^′^). Once the mean (a zero matrix) and **K** are established, we can sample from the prior distribution for ***f***(**x**) (see Texts S1 and S6 in the Supporting Information for details).

Sampling every possible value of the function ***f***(**x**) across a continuous domain is not practical. Instead, we sample a finite set of points, leading to a vector of function values, ***f*** = {*f*(**x**_1_), *f*(**x**_2_), ..., *f*(**x**_*N*_)}, which follows a joint Gaussian distribution with a mean vector **μ** = *m*(**x**_1_, **x**_2_, ..., **x**_*N*_) and covariance matrix **K**_*i*,*j*_ = *k*(**x**_*i*_, **x**_*j*_). In this work, the terms “kernel” and “covariance function” are used synonymously to refer to the function that defines the covariance between any two points in the input space. We describe the mean and covariance functions in the next section.

We demonstrate a basic GP model to help readers understand its operation through GPyTorch implementations (Figure 1). GPyTorch offers a robust and adaptable GP framework that benefits from accelerated computation through GPU support.^18^ Although GPyTorch was not originally developed as a PPL in the strict sense, it integrates seamlessly with the Pyro PPL (https://pyro.ai). Therefore, we consider it a PPL based on its actual functionality. Figure 1a shows 10 samples (i.e., 10 sampled trajectories, each depicted as a curved line) from the prior distribution of the GP function, chosen with a relatively short length scale (i.e., 1 day) for the kernel. The prior sampling in the input space was conducted over the measurement times corresponding to OCO-2/3 observations (a total of 71 data points), which were aggregated at the GC modeling resolution of 0.5° × 0.625° for July 2020. Therefore, the spatial dimension was not considered for the kernel in this example, but it was included in the complete inference work later (see Section 2.1.2). Figure 1b illustrates a GP function characterized by a longer length scale (i.e., 7 days), indicating that the values of the function at two distinct points remain correlated across larger distances, leading to more gradual variations within the function.

The key distinction of GPs from many models, including the traditional inverse models, is that they establish priors over entire functions rather than individual parameters.^31^ This means the GP provides a probability distribution over all possible functions that could fit observations. For instance, in Figure 1a,b, we display just 10 possible samples of the function from the prior distribution, which could fit observations. Figure 1c illustrates how the prior distribution of the function ***f*** is influenced by four observations, akin to how traditional inversion models use observations to estimate posterior emissions. In this figure, we show a GP model with posterior uncertainty (2σ) and four observations (red dots) used to predict the function ***f*** over the input space *X* (with 71 prediction points and a length scale *l* = 7.7 days). In this case, the GP is conditioned on the four known data points (i.e., the training data consists of four points), using them to inform the joint distribution over the 71 prediction points. In Figure 1c, each realization of the function is represented by a gray line (100 samples shown), which is used to estimate the 2σ confidence intervals. In Figure 1c, an arbitrary point at *X* = 0.536 (green vertical line) is selected from the input space. We “slice” the GP prediction at that point and rotate it, which is shown in Figure 1d. Then, this rotation represents a conditional distribution derived from “slicing” the GP at *X* = 0.536. Each ***f*** value on the *X*-axis of Figure 1d corresponds to the function value evaluated at *X* = 0.536, which is represented by the gray line in Figure 1c and is used to calculate a probability density.

#### 2.1.2 GP for Flux Inference

In this section, we describe how GP models can be used to estimate CO_2_ fluxes from OCO-2/3 satellite observations. We use GP modeling in a regression analysis setting because it offers two key advantages: the ability to specify prior distributions for hyperparameters and to characterize complex spatiotemporal covariance structures in satellite CO_2_ observations. The GP kernels capture several important sources of covariance in our data: (1) temporal variations in flux patterns, such as those driven by synoptic-scale weather patterns (operating over periods of several days),^32^ (2) spatial correlations in emission fluxes that reflect underlying patterns in land cover, (3) autocorrelation in satellite measurements taken over the same location at different times, (4) cross-correlations between different emission sectors across space and time, and (5) systematic model errors, particularly from transport modeling, which tend to show spatial and temporal correlation patterns.^33^ Our GP framework allows us to explicitly account for these various correlation structures while maintaining the flexibility to discover patterns directly from the data.

To demonstrate why this complex approach is necessary, we conducted a simple linear regression analysis using the same data set. As shown in Figure S1, the linear regression coefficients deviate from the true scaling factors. Most critically, for the fire sector, the linear regression produces a physically implausible negative coefficient, suggesting that increased fire emissions would decrease atmospheric CO_2_ concentrations—a result that violates fundamental atmospheric physics. In the GP framework, we prevent such physically implausible results by incorporating domain knowledge through prior distributions for the parameters and capturing the inherent spatiotemporal relationships through the GP kernel.

The two key components of a GP model are the mean function and the kernel (see eq 1). For a flux inference application, we define the mean function *m*(**x**), which is compared with the noisy version of the target variable (e.g., OCO observations), as
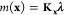
3where **K**_**x**_ represents the input data set from the GC transport model, which is based on our prior emissions estimates and is structured as an *n* × *k* matrix (*n* = number of data points (i.e., 71) and *k* = 4 (i.e., 4 sectors); refer to Section 2.4 for details), and λ (*k* × 1), a GP hyperparameter vector, signifies a set of scaling factors to be inferred from the data. These scaling factors, which are our primary state vector of interest, are crucial for aligning our prior emissions with observations. This mean function approach is broadly embraced in the atmospheric inverse analysis field for estimating greenhouse gas (GHG) emissions.^8,10,30,34^ Note that as described, this mean function is linked to the GP function ***f***(**x**), not directly to the target variable ***y*** (see eqs 1 and 2). This is a key distinction from traditional linear inverse models, which are represented by ***y*** = **K**_**x**_λ + ϵ.

The second component of a GP model is the covariance function, or GP kernel, which defines the relationships between function values at different points (e.g., OCO observation locations in time and space). We need to construct kernels to represent the spatiotemporal characteristics embedded in OCO observations. For instance, weather patterns might persist for several days. In such cases, our GP model employs a temporal kernel to link observations that occur close in time, assuming they likely share similar weather conditions. Similarly, a spatial kernel is used to connect observations based on their geographical proximity. For example, this kernel captures the relationship between observations made within a short distance of each other in the same urban area or forest. By doing so, the model effectively captures and interprets the underlying spatial and temporal covariance present in real-world CO_2_ data. This helps predict CO_2_ levels based on both where and when measurements occur, allowing us to generate more accurate and contextually relevant results from the data.

The spatiotemporal kernel matrix is created by multiplying the spatial and temporal kernels
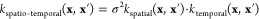
4where σ^2^ denotes the variance of the kernel, which scales the amplitude of the function values predicted by the GP, and the spatiotemporal kernel, *k*_spatio-temporal_, is realized by element-wise multiplication of the spatial, *k*_spatial_, and temporal, *k*_temporal_, kernels. The resulting spatiotemporal kernel maintains the dimensionality of its constituent kernels. In this work, all three kernels in eq 4 yield a covariance matrix of size 71 × 71, corresponding to the 71 unique observations of the OCO-2/3 satellite in both spatial and temporal dimensions.

For our analysis, we selected widely used kernel functions: the Matérn 5/2 kernel for spatial data modeling^35^ and the squared exponential kernel for temporal correlations. However, we have not examined the impact of different kernels on characterizing temporal, spatial, or spatiotemporal correlations. Testing various kernels for satellite CO_2_ observations, including the development of custom kernels, merits further studies in GP-based inverse modeling. The detailed forms of the spatial and temporal kernels and the prior distributions for the kernel parameters are provided in Texts S1 and S2, respectively.

### 2.2 Prior Emissions

In the Bayesian model perspective, prior emissions represent our a priori knowledge of the emission fluxes to infer unknown true emissions. We constructed prior emissions (in flux units) for four sectors: fossil fuel (FF; including cement production), net ecosystem exchange (NEE), which is the difference between ecosystem respiration and gross primary production, fire, and ocean. All emissions were gridded from their native resolutions to the CALGEM (California Greenhouse Gas Emission Measurements) domain,^23,36^ which covers 20°N to 59.9°N and 130°W to 105.1°W at 0.1° × 0.1° resolution. The emissions were constructed at hourly temporal resolution. These emission maps were further aggregated into 0.5° × 0.625° resolutions to be used as the input to the GC CTM (see Section 2.3) to calculate column average concentrations of CO_2_.^28,29^

FF CO_2_ emissions were prepared by integrating 1 km hourly emission data from Vulcan 3.0^37^ with estimates from the California Air Resources Board (CARB). To create a spatial inventory for California in 2020, we applied scaling factors derived from CARB’s 2020 data to the most recent 2015 Vulcan data. The scaling factors applied to each sector are listed in Table S1. To account for the diverse changes in emissions resulting from COVID-19 pandemic lockdowns, we adjusted FF CO_2_ emissions for those sectors that were excessively impacted by the restrictions. A detailed explanation of these adjustments can be found in Text S3. Also, we describe the prior fluxes for the NEE, fire, and ocean sectors in Text S4.

**Figure 2:**
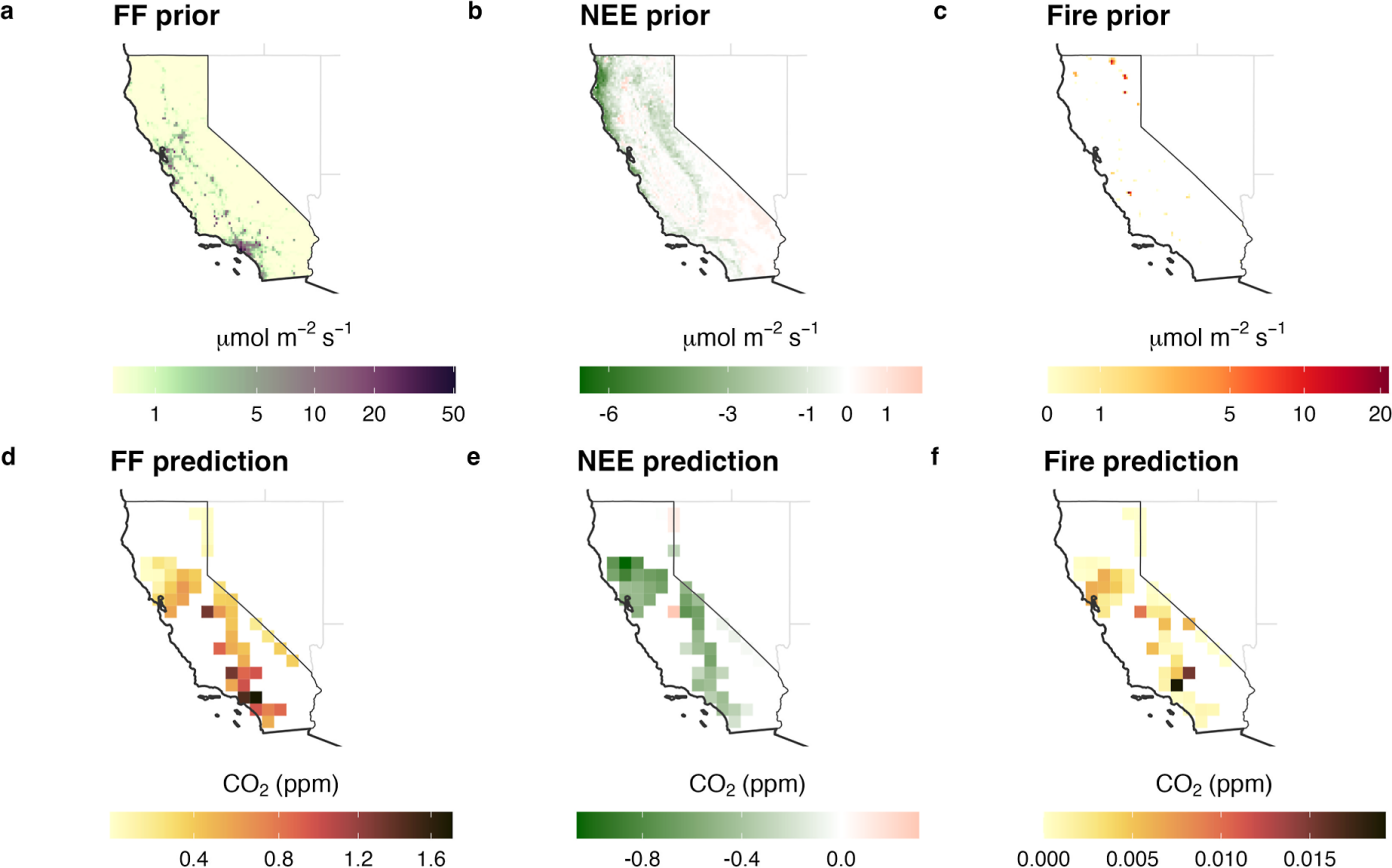
Average prior CO_2_ emission flux (0.1° × 0.1°) maps and GC-predicted monthly average concentrations (i.e., XCO2 enhancement; 0.5° × 0.625°) for July 2020: (a) FF prior, (b) NEE prior, (c) fire prior, (d) FF prediction, (e) NEE prediction and (f) fire prediction.

Figure 2a–c present the mean emissions for the FF, NEE, and fire sectors (excluding ocean-related fluxes) during July 2020, which is the study period for this work (also, see Figure S2 for California’s land cover types). FF emissions predominantly emanate from the main urban regions, while onroad emissions are discernible along California’s extensive highway system (see Figure S2). NEE fluxes display a smoother variation across different land types compared to FF emissions. As expected, forested regions exhibit negative fluxes, indicating carbon uptake, whereas urban and barren landscapes tend to have positive fluxes, reflecting net carbon release. The fire emissions map for July 2020 shows varied biomass-burning emissions across California. Despite major fires occurring later in August and September, some local areas in July are estimated to emit fire emissions comparable to FF emissions from California’s urban areas.

### 2.3 Transport Modeling

The GC CTM, version 14.0.1, was used to determine the atmospheric levels of CO_2_.^28,29^ A simulation for July 2020 was conducted for the North American domain, spanning from 10° to 70°N latitude and 40° to 140°W longitude, driven by MERRA-2 meteorology.^38^ This simulation was performed on a horizontal grid resolution of 0.5° × 0.625° and covered 47 vertical levels from the surface to 0.01 mb. The CO_2_ boundary conditions for the nested North American simulations were provided by the global GC model employing a 4D-Var data assimilation system, which operates globally at a coarser 4.0° × 5.0° horizontal resolution over the same vertical levels. These global CO_2_ simulations were refined using inverse modeling techniques that incorporated XCO2 observations from the OCO-2 satellite’s land nadir and land glint modes, as well as global in situ observations from 2015 to 2020.^39,40^ Additional information on source-attributed simulations is described in Text S5.

Figure 2d–f show the GC predictions (monthly averages) of XCO2 for FF, NEE, and fire (biomass burning) sectors, aligned with the observations from the OCO-2/3 satellite for July 2020. The FF-related XCO2 estimates indicate pronounced concentrations over Southern California, highlighting carbon emissions from the mega urban area. The NEE-derived XCO2 shows negative values across the state, which is consistent with the expected summertime carbon uptake by ecosystems. For the fire sector, the monthly average predicted XCO2 highlights areas of biomass burning in California, as shown in the fire emission map (Figure 2c). It is important to note that not all OCO measurements coincide with the grid cells where active fires were detected.

### 2.4 GP Modeling Set-Up and Implementation

A primary objective of this work is to evaluate the GP model’s efficacy in retrieving the true scaling factor vector, **λ**_true_ (unknown to the GP machine), from input data that has been inversely scaled by **λ**_true_. For this purpose, we consider raw GC-simulated total XCO2 as our target variable (***y***; akin to observations in an atmospheric inversion) within an ML framework. Thus, we do not directly use OCO-2/3 observations as the target variable; instead, their observation times and locations serve as the input data in our GP modeling. To construct **K**_**x**_ matrix in the GP mean function (eq 3), we apply the inverse of scaling factors (0.7, 0.6, 1.2, and 0.9) to the GC predictions for the FF, NEE, fire, and ocean sectors, respectively. This adjustment is equivalent to setting λ_true_ = [0.7, 0.6, 1.2, 0.9] as per eq 3. The FF column in the **K**_**x**_ matrix, for instance, is produced by multiplying the original GC prediction by the reciprocal of its corresponding scaling factor (1/λ_FF_ = 1/0.7).

We implement inverse modeling through three distinct methodologies: (1) fully Bayesian GP (FBGP; our primary GP model), (2) GP using marginal log-likelihood optimization (GP MLL; see https://sites.google.com/lbl.gov/calgem/GP for the implementation), and (3) the classical Bayesian (CB) method, which is based on an analytical solution^41^ and widely adopted in prior studies.^36,42,43^ While FBGP treats each hyperparameter as a random variable (i.e., described by a probability distribution), the GP MLL optimization in our base case finds the single best set of hyperparameters (i.e., point estimates) that maximizes the likelihood of the data under the GP model. We also present an additional analysis in which we estimate the uncertainty for the GP MLL approach (see Section 3.3). We employ the PyMC PPL^44^ for implementing the FBGP, and GPyTorch PPL^18^ for the GP MLL optimization. To help readers understand marginalization in the context of GP, we introduce details on GP MLL in Text S6 of Supporting Information.

The target (***y***) is a noisy version of the unknown true function, as shown in eq 2. We assign a noise of 0.5 ppm (unknown to the GP machine as a hyperparameter) in the form of a standard deviation in its sampling distribution. The concentration of 0.5 ppm is ∼30% of the maximum total concentration in Figure 2 and is within the OCO-3 instrument error level of 0.23–2 ppm^4^.

A Markov chain Monte Carlo (MCMC) algorithm is applied to estimate the GP’s hyperparameters in the FBGP approach. MCMC methods have been applied to atmospheric inverse modeling studies.^10,20,23,25,45^ In this analysis, we apply a more recent MCMC algorithm, the No–U-Turn Sampler (NUTS),^46^ to a GP model. The NUTS algorithm is a recent extension of another popular MCMC algorithm, Hamiltonian Monte Carlo (HMC). HMC is known to estimate probability distributions more effectively than traditional MCMC algorithms by using principles from physics to explore the parameter space.^47^ Thus, we implement the FBGP approach using a modern MCMC algorithm, NUTS, and the modern PPL, PyMC (Version 5.10.3).

## 3 Results and Discussions

### 3.1 Prior and Posterior Predictive Checks

**Figure 3:**
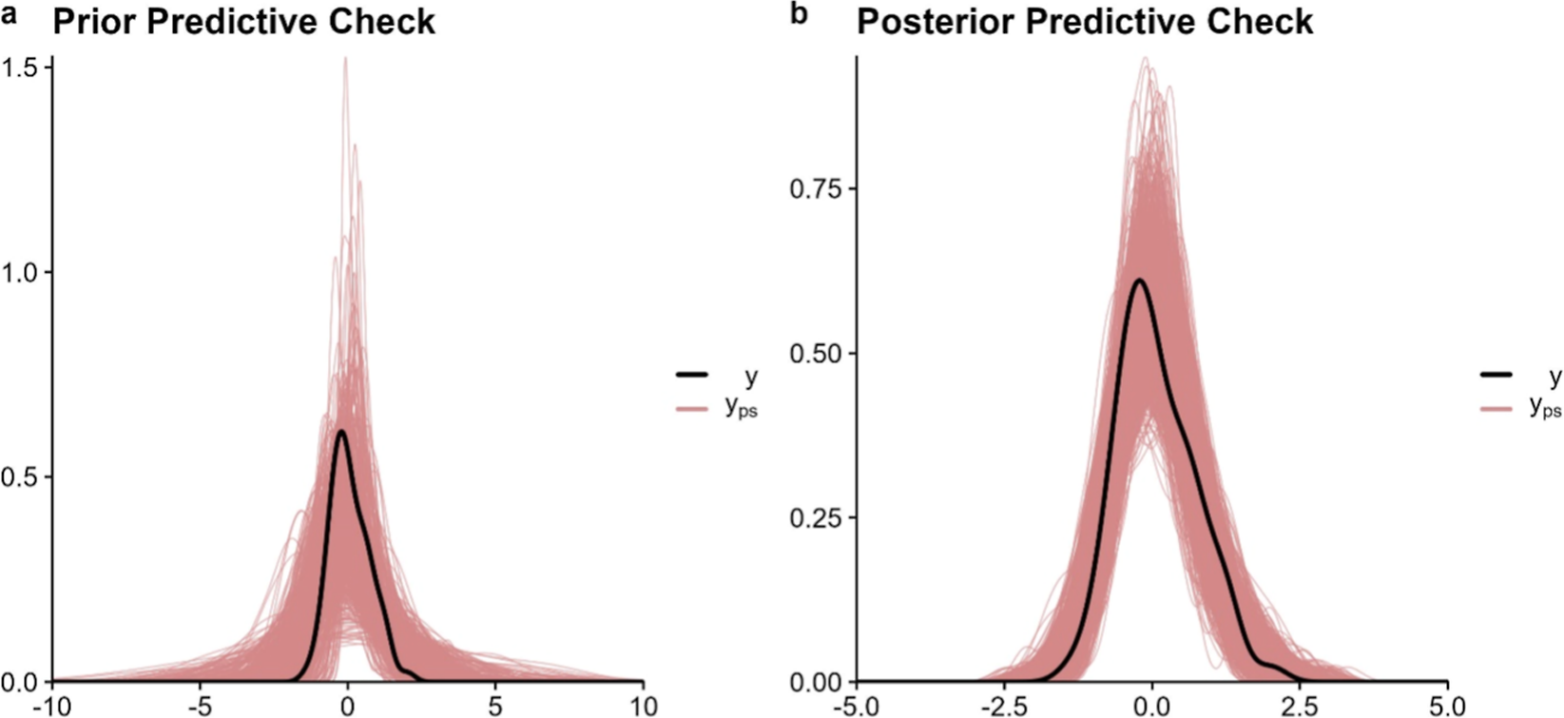
Prior (a) and posterior (b) predictive checks from the inversion using probability density functions. *y* and *y*_ps_ represent observations and predictive samples, respectively. The simulated data were generated by the PyMC PPL.

In Bayesian modeling including a GP model, a prior predictive check is a method used to validate the choice of priors before fitting the model to the observed data. This process helps in assessing whether the prior distributions make sense given our knowledge of the expected outcome (here XCO2) and can lead to realistic modeling in the posterior estimates.^48^ For example, vague priors may not incorporate our domain knowledge of atmospheric CO_2_. Practically, a prior predictive check involves initially sampling parameter values from their respective prior distributions. Subsequently, data (e.g., XCO2) is sampled from the likelihood specified by the model based on these sampled parameters. In this work, we conduct the prior predictive check using the PyMC PPL, which provides convenient functions for this purpose. Figure 3a shows the prior predictive check based on our model and prior distributions (see Figure S3 and Text S2 for prior distributions for the hyperparameters) using a probability density function (PDF) plot. In the figure, the prior predictive simulations generate data that broadly encompass the observed data, suggesting that the prior distributions are reasonable.

The posterior predictive check (PPC) is similar to the prior predictive check. In Bayesian modeling, we update our prior knowledge (i.e., prior distribution) using observed data. The idea of PPC is to utilize the posterior distribution of the model parameters to generate new data sets and then compare these new simulated data sets to the actual observed data.^48^Figure 3b displays a PDF plot of the PPC result, demonstrating that the samples realized from the posterior parameters effectively encompass the observed data. This result indicates that our posterior parameters are representative of the underlying data distributions. Also, Figure 3 shows that the PDF for the PPC is much tighter than that of the prior predictive check, as expected.

Our prior and posterior predictive checks advance beyond previous studies that primarily examined goodness-of-fit metrics, such as the alignment of slopes between posterior estimates and observations. While these previous approaches relied on summary statistics, our PPC approach generates predictions from the posterior distributions of parameters, enabling comprehensive model validation. After training our model with observational data to obtain posterior parameters, we use these optimized parameters to generate new predictions and compare them against actual observations to assess if the model adequately captures the underlying data-generating process. This approach provides more rigorous validation because PPC tests the model’s ability to generate realistic data by examining the full probability distribution of predictions (as shown in Figure 3), rather than just fitting existing data and summarizing results. For example, a model might show acceptable summary statistics (e.g., low root-mean-square error) while still generating physically implausible values or failing to capture important characteristics of the system.

### 3.2 HyperParameters and Covariance

Hyperparameters play a crucial role in ML models, including GPs.^49,50^ GP hyperparameters include those from the kernel, the mean function, and the likelihood. These hyperparameters control the behavior of the learning algorithm and significantly impact the model’s performance. For example, the length scale parameter controls the smoothness of the GP functions (see Figure 1). Thus, these hyperparameters are critical components to capture the spatiotemporal correlation among observations.

**Figure 4:**
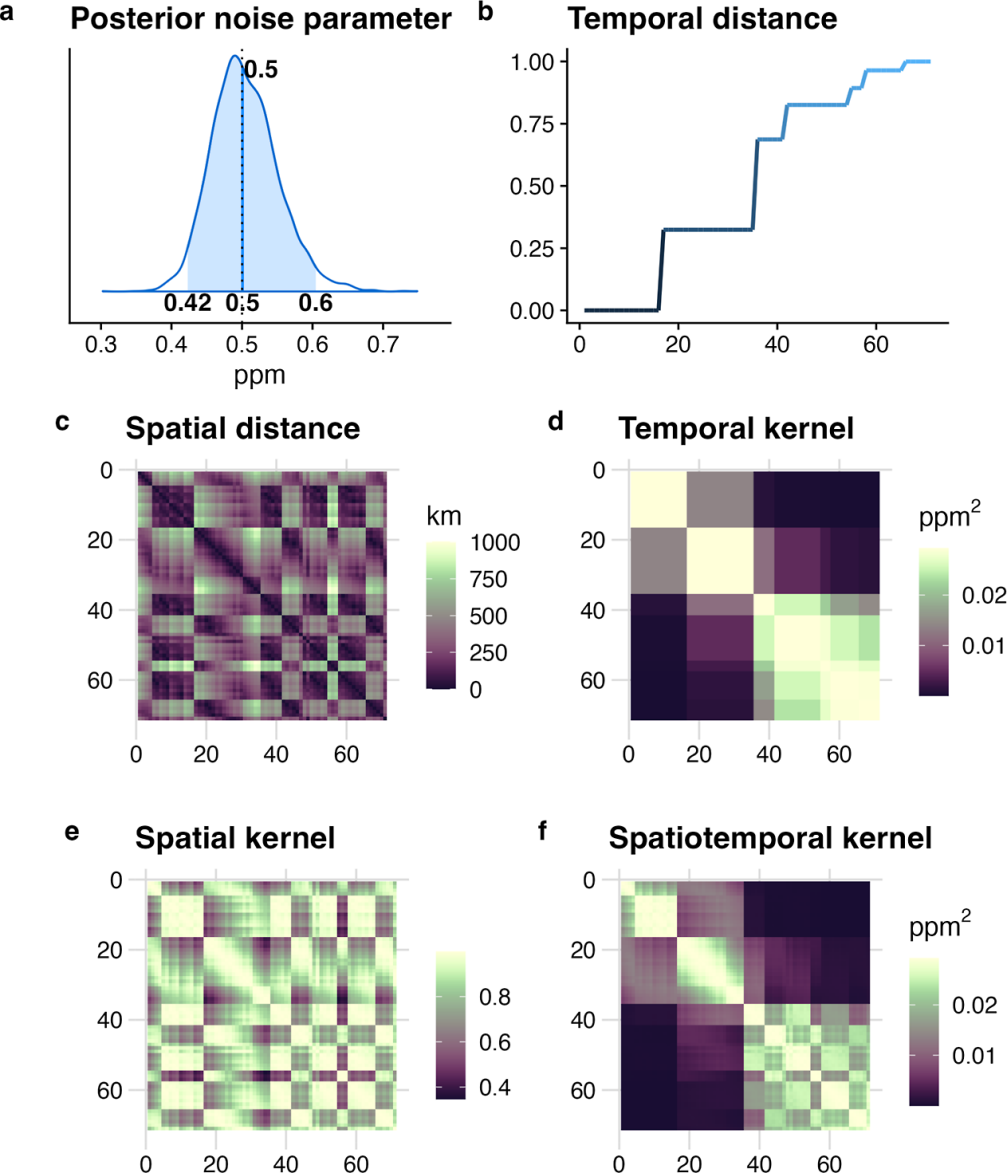
Estimated noise parameter, temporal and spatial distances, and kernels: (a) estimated PDF for the noise parameter, (b) normalized temporal distance, (c) spatial distance, (d) temporal kernel, (e) spatial kernel, and (f) the combination of the temporal and spatial kernels. We multiplied the kernel variance (σ^2^) by the temporal kernel, resulting in units of ppm^2^.

Figure 4 shows the posterior noise parameter and the kernels based on the parameters we estimated using the FBGP approach (implemented by PyMC). As described in Section 2, the true noise was a standard deviation of 0.5 ppm, and the PDF plot shows that the FBGP model successfully recovered it with a median value of 0.5 ppm. The essential data for constructing the kernel is the distance between data points in time and space. Figure 4b,c show the temporal (normalized) and spatial distances, respectively, which are used to construct the temporal and spatial kernels in Figure 4d,e. The normalized temporal distance in Figure 4b indicates that the OCO data are available every few days, resulting in a stepwise monotonic increase. On the other hand, the spatial distance in Figure 4c illustrates the variation in distances between observations, which cycles due to data being available every few days. Figure 4f shows the spatiotemporal kernel, which combines the temporal kernel from Figure 4d and the spatial kernel from Figure 4e.

These spatial and temporal kernels fundamentally shape the model’s covariance structure and consequently its performance, such as correctly estimating the scaling factor. Specifically, the spatial kernel determines how the model interpolates between observation points, with different interpolations resulting in different posterior parameter values. As described in Section 2.1, this kernel’s hyperparameters, particularly the length scale, control the smoothness assumptions of the spatial field and directly impact the model’s ability to resolve fine-scale emission patterns while avoiding unrealistic spatial discontinuities. The temporal kernel captures both regular patterns (such as synoptic and seasonal cycles in emissions) and irregular temporal variations. The parametrization of this kernel affects how the model handles temporal autocorrelation and determines its sensitivity to both gradual trends and abrupt changes in emission patterns.

### 3.3 Estimation of Scaling Factor Parameters

**Figure 5:**
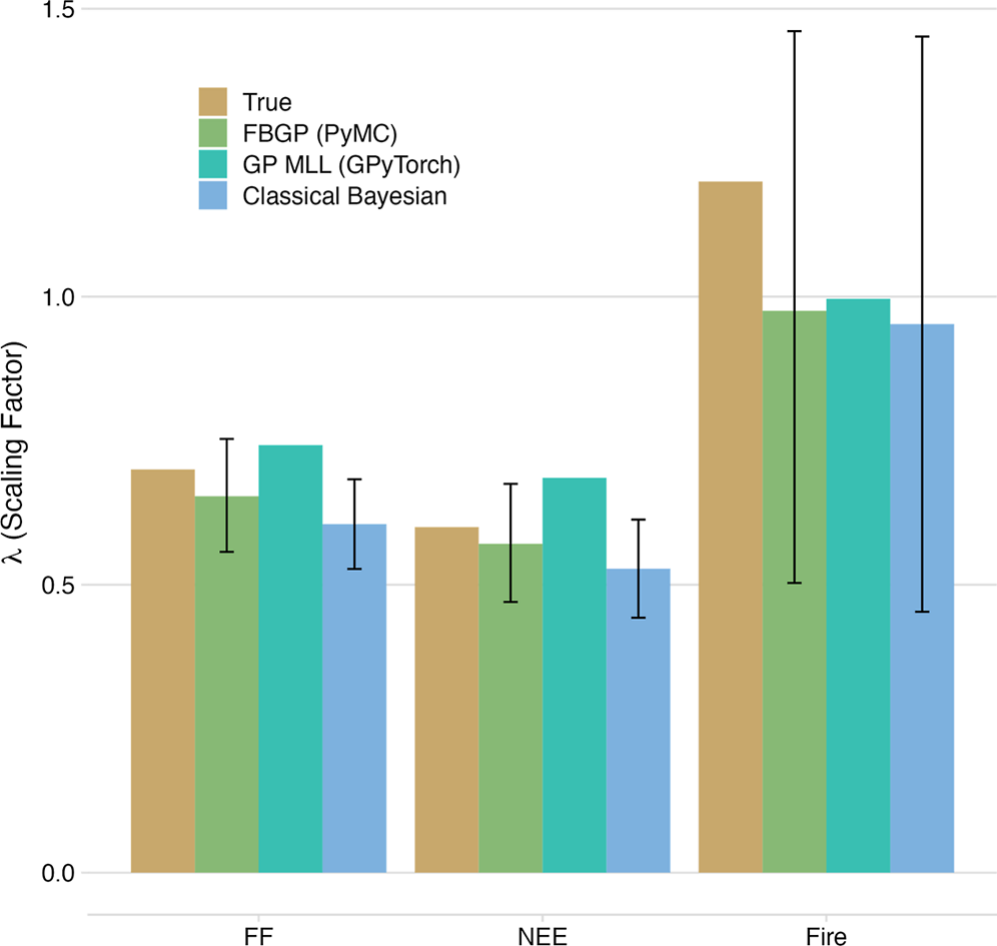
Comparison of true and inferred scaling factors by sector between FBGP, GP MLL, and CB. Error bars represent the 68% confidence intervals.

In this section, we demonstrate the key capability of our GP models to accurately retrieve the true parameter values of the scaling factors, which constitute the state vector of interest in atmospheric inverse modeling. Recall the scaling factors are the hyperparameters used in the mean function, *m*(**x**) (eq 3). As described, we conducted inverse modeling using three different approaches: (1) FBGP, (2) GP MLL optimization and (3) the CB method (Section 2.4). In Figure 5, we present the true values of the scaling factors, **λ**_true_, which are 0.7, 0.6, and 1.2 for the three major sectors of FF, NEE and fire, respectively.

Figure 5 demonstrates that the FBGP approach accurately identifies the true scaling factors for all three sectors within the 68% confidence bounds. The FBGP model closely approximates the true values for the FF and NEE sectors, while the fires sector exhibits greater uncertainty. This disparity is due to the more substantial XCO2 contributions from FF and NEE compared to fires, with the lesser XCO2 signal (relative to the noise) from fires leading to lower certainty in the posterior estimates.

The GP MLL approach also estimates scaling factors that closely align with the true values, as demonstrated in Figure 5 (see Text S7 for the GP MLL implementation details). As shown in Figure 5, we present the central estimates of the scaling factors using the GP MLL method, highlighting its significantly faster performance in estimating these values compared to the FBGP method (refer to Section 3.4 for details on the utility of GP MLL). Furthermore, the uncertainty estimates for the GP MLL method are computed using the fisher information matrix (FIM), which represents the covariance of the gradient of the log likelihood.^15^ In this study, we employ the automatic differentiation capabilities of GPyTorch to calculate this gradient. A detailed description of the FIM approach to estimating uncertainties in GP MLL is provided in Text S8, and the results are displayed in Figure S4. We note that GPyTorch is capable of full Bayesian modeling, including uncertainty quantification through the incorporation of another probabilistic programming language (i.e., Pyro; https://pyro.ai/). GPyTorch’s MLL optimization is significantly faster than FBGP and is useful for quickly obtaining the central estimate (see Text S9 and Figure S5 for details on the computation cost comparison, and refer to Section 3.4 for further discussion). To the best of our knowledge, this is the first implementation of the GP MLL approach, demonstrating that modern Bayesian ML optimization, specifically through the use of training data sets, can effectively infer GHG emissions. Utilizing GPyTorch, the GP MLL approach shows that modern ML methods and platforms are viable for atmospheric inverse modeling.

To assess the robustness of our posterior estimates, we performed the inversion 10 times for both the FBGP and GP MLL methods (see Figure S6). The posterior scaling factors demonstrated strong convergence across all iterations for both methods, with minimal variation in the estimates. This consistent convergence suggests high reliability in our inversion results.

The CB (classical Bayesian) method is used as a reference to compare with the GP approaches (see Text S10 for a detailed description of the CB method). The CB method generally recovers the true scaling factors (Figure 5). However, a key challenge with the CB method based on the analytical solution is estimating the noise parameter, as there is no straightforward method for its estimation (see Section 3.4 for more discussion). For this reason, we used the true noise parameter value (i.e., 0.5 ppm) in the CB method to focus on the inference of the scaling factors. Using the true noise parameter, we constructed a diagonal covariance matrix (without the off-diagonal term), which has been widely used in atmospheric inversion studies.^30,33,42,51^ We offer suggestions for a more accessible approach to estimating hyperparameters, including the noise parameter, in Section 3.4.

Although our work’s primary goal is to test the capability of the GP-based inversion to retrieve unknown parameters (such as scaling factors for emission adjustment), we also conducted an inversion using actual OCO-2/3 observations from July 2020, the same period as our original analysis. The results are presented in Text S11 and Figure S7 in the Supporting Information.

### 3.4 Implications for Future Inverse Modeling

We demonstrated that the GP model, when applied to GC model predictions for OCO observations, successfully separated sector emissions by accurately recovering the true scaling factors. This indicates that GP models can serve as a powerful ML framework for inferring GHG emissions. Also, we illustrated how the GP models effectively captured the spatiotemporal correlation structure inherent in OCO satellite observations. Specifically, we captured the spatiotemporal correlation entirely within the kernel function of the Gaussian process model. This kernel integrates both spatial and temporal dependencies by modeling how observations relate to each other based on their distances in space and time. This approach allows us to represent the influence of CO_2_ concentrations at specific locations and times on nearby observations, effectively accounting for natural diffusion and transport processes in the atmosphere.

The FBGP approach demonstrated that these hyperparameters can be simultaneously estimated alongside the state vector of the scaling factors.^10,23,25,45^ Future inverse studies are likely to benefit from our FBGP approach, which provides a robust method for inferring all hyperparameters—instead of prescribing them or using ad-hoc methods—as demonstrated by the PPC result in Figure 3 and the noise parameter estimation in Figure 4.

Our findings show that the FBGP model outperforms the GP MLL model in accurately estimating these scaling factors (λ) with associated uncertainty and in effectively revealing hidden noise levels in the data. However, the GP MLL model still presents significant benefits and potential for future atmospheric inverse modeling. To the best of our knowledge, this work represents the first application of a traditional ML approach—specifically, a training-validation framework for GP MLL—to estimate CO_2_ emissions by optimizing scaling factors for emission adjustments (see Text S7 for details). Our work highlights the advantages of the ML approach within the GP model, particularly its capacity for rapid validation of model robustness. This is a critical asset as satellite data sets expand and their spatiotemporal covariation structures become increasingly complex and computationally demanding. While the GP MLL model may not provide parameter estimates as robust as the FBGP method, it remains a valuable method for quickly assessing the robustness of the inverse model compared to more computationally intensive FBGP models. In Text S7, we describe one of the possible implementations of the GP MLL approach. However, our findings suggest that exploring different implementations in future work could enhance the inference capabilities of the GP MLL approach for both the central and uncertainty estimates (see Text S8 for details on the uncertainty estimation for GP MLL).

We showed how prior predictive checks can be utilized to validate our prior assumptions regarding the parameters, such as the prior uncertainty for the scaling factor. Similarly, we employed PPCs to validate the optimized parameters, including the scaling factors. This involved generating posterior samples for the target variable, which is the CO_2_ concentration in our case, from posterior hyperparameter samples (Figure 3). Despite the importance of validating both prior assumptions and posterior estimates, this process is often overlooked in atmospheric inversion studies. Modern PPLs such as PyMC—which was used in this work—and Stan^52^ (another widely used PPL) offer tools to facilitate these checks although they can also be manually performed using samples from the prior and posterior distributions of the hyperparameters, as described briefly in Section 3.1. We recommend routinely incorporating prior and posterior predictive checks in atmospheric inverse modeling to enhance model reliability and ensure accurate representation of underlying processes.

The computational cost required to run the GP inversion can be substantial, especially for large data sets or complex models, due to the cubic scaling with the number of data points *O*(*n*^3^).^14^ When employing a full Bayesian treatment, as in our FBGP model using algorithms like NUTS, the computational cost can increase significantly due to the numerous iterations required for convergence. However, modern hardware such as GPUs can significantly reduce computational time. We provide a detailed comparison of computational costs between FBGP and GP MLL across different platforms (CPU and GPU) in Texts S9 and S12, and Figures S5 and S8.

The atmospheric inverse modeling community can benefit from the recent advancements in HPC to implement complex ML models,^53^ such as GPs, which have previously been limited by computational costs. Our results indicate that the GP model can potentially be applied on much larger spatial scales. For an example of future applications to larger spatial scales, we modeled the covariance structure (Figure S10) corresponding to OCO-3 observations from July 2023 (Figure S9). This example demonstrates how the spatiotemporal covariance structure differs from the typical temporal or spatial covariance, underscoring the necessity to incorporate both dimensions. Further details are presented in Text S13 of the Supporting Information.

In atmospheric inverse analysis, many studies employing the CB method use the term “model-data mismatch” to represent the error term. For the error term in the CB method, some studies prescribe the error as a fraction (e.g., 30%) of the mean observation, while others estimate the error term by explicitly considering uncertainty sources such as wind and planetary boundary layer errors, which require significant efforts to characterize.^51^ It can also be derived from reported errors in the literature,^4^ which do not guarantee its applicability to specific inversion applications. Other studies employed maximum likelihood estimation (MLE) or similar methods.^54,55^

Although MLE (or similar methods) can be used, deriving a closed-form solution for MLE is challenging for most atmospheric inverse modelers as the complexity of the inversion model increases. Also, in many cases, the MLE method is a sequential approach (for convenience) where parameters such as the noise (σ_noise_^2^) are estimated separately from the scaling factor (λ). For example, we first estimate σ_noise_^2^ by maximizing the likelihood function, focusing only on this parameter, and possibly using residuals from an initial estimate of λ based on a default σ_noise_^2^; estimating λ is done in the next step. On the other hand, MCMC (e.g., HMC–NUTS in this study) captures the dependencies between parameters, which might be missed when estimating them separately as in MLE. For example, the uncertainty in σ_noise_^2^ affects the estimation of λ, and vice versa. Thus, MCMC offers a more robust approach to parameter estimation in atmospheric inverse modeling by estimating all parameters simultaneously, albeit at the cost of higher computational demand. However, we note that improvements in software (e.g., JAX) and hardware (e.g., GPU) performance will likely reduce the computational cost, as demonstrated in this study (see Text S12).

Our work demonstrated the potential of GP models to enhance the accuracy and efficiency of atmospheric inversion modeling that assimilates satellite observations of GHGs. Our GP framework holds potential for other major GHGs, such as methane (CH_4_) and nitrous oxide (N_2_O), which exhibit more distinct emission characteristics than CO_2_. For instance, CH_4_ emissions often originate from more localized sources like oil and gas production or agricultural (e.g., dairy farms) activities, presenting marked spatial and temporal variability compared to the relatively smoother patterns observed in CO_2_ emissions. To address these differences, it is necessary to adapt the covariance structures of GP models. Our framework’s inherent flexibility allows for the modification of kernels to better capture the complex dynamics of CH_4_ and N_2_O emissions. For example, kernels can be manipulated—either multiplied, added, or both—to represent the spatiotemporal characteristics of the greenhouse gas of interest.

Our method holds significant promise for enhancing the precision and scalability of regional GHG emission monitoring programs, particularly when utilizing satellite or multitower observations. It can be integrated into existing air quality frameworks (e.g., for monitoring carbon monoxide emissions from wildfires) and climate policies (targeting other GHG species), thereby improving the near real-time tracking of GHG and wildfire emissions through advanced inference models and enhanced computational capabilities. For instance, in California, this capability is crucial for meeting the state’s ambitious climate targets, which demand rigorous and timely assessments of emission sources and reductions across diverse economic sectors. As demonstrated in our analysis, the kernel-based GP method can seamlessly incorporate data from multiple towers in CARB’s GHG measurement network, satellite data, or a combination of remote sensing and ground observations, and it can be adapted to other regions of the country with similar observation networks.

Furthermore, our methodology could support national and international climate policy frameworks by providing more accurate estimates for CO_2_ and other emissions, using globally available satellite or ground-based network observations. Policymakers can thus better assess compliance with emission reduction commitments and adapt mitigation strategies more effectively. The challenges posed by larger-scale computations can be addressed through GPU-based computing, parallelization, and other technologies such as quantum computing. We anticipate that this aspect of the method will benefit from rapidly evolving hardware and software developments.

## Supporting Information Available

Description of Gaussian process kernels, probability distribution of prior parameters, methods for prior emissions, details of the GC model simulations, details of the GP MLL and classical Bayesian methods, uncertainty estimation method for GP MLL, and details of the computational cost for GP inverse modeling (PDF)

## Abbreviations

CALGEM: California greenhouse gas emission measurements
CARB: California air resources board
CB: classical Bayesian
CTM: chemical transport model
FBGP: fully Bayesian Gaussian process
FF: fossil fuel
GC: GEOS-Chem
GHG: greenhouse gas
GP: Gaussian process
GPU: graphics processing unit
HMC: Hamiltonian Monte Carlo
HPC: high performance computing
MCMC: Markov chain Monte Carlo
ML: machine learning
MLE: maximum likelihood estimation
MLL: marginal log likelihood
NEE: net ecosystem exchange
NUTS: no–U-turn sampler
OCO: orbiting carbon observatory
PDF: probability density function
PPC: posterior predictive check
PPL: probabilistic programming language.
