# Supplementary material for: Applying Gaussian Process Machine Learning and Modern Probabilistic Programming to Satellite Data to Infer CO2 Emissions
Source: Environ Sci Technol. 2025 Feb 24;59(9):4376–87. doi: 10.1021/acs.est.4c09395 (PMC11912316; doi:10.1021/acs.est.4c09395)
Supplement: Supplementary file 1 — es4c09395_si_001.pdf [file es4c09395_si_001.pdf]

Supporting Information

Applying Gaussian Process Machine Learning and Modern Probabilistic Programming to Satellite Data to Infer CO_2 Emissions

Seongeun Jeong[1,], Sofia D. Hamilton[1], Matthew S. Johnson[2], Dien Wu[3], Alexander J. Turner[4], Marc L. Fischer[1]*

[1]Energy Analysis and Environmental Impacts Division, Lawrence Berkeley National Laboratory, Berkeley, CA 94720 USA

[2]Earth Science Division, NASA Ames Research Center, Moffett Field, CA 94035, USA

[3]Division of Geological and Planetary Sciences, California Institute of Technology, Pasadena, CA 91125, USA

[4]Department of Atmospheric and Climate Science, University of Washington, Seattle, WA 98195, USA

[*]Correspondence: Seongeun Jeong (sjeong@lbl.gov)

This 30-page SI file (in PDF) includes:

- Supplementary Texts S1 to S13
- Tables S1 to S2
- Figures S1 to S10
- References

Text S1. Spatial and Temporal Kernels

For the spatial analysis in our study, we employ the Matérn 5/2 kernel, a popular choice within the Matérn family of covariance functions for spatial data modeling. This family is favored for its ability to control the function's smoothness flexibly[1]. The Matérn 5/2 kernel between two spatial points can be expressed as:

$$k_{\text{spatial}}(\mathrm{x}, \mathrm{x}') = \left(1 + \frac{\sqrt{5}r}{\ell_s} + \frac{5r^2}{3\ell_s^2}\right) \exp\left(-\frac{\sqrt{5}r}{\ell_s}\right)$$

$$r = \sqrt{(x_1 - x'_1)^2 + (x_2 - x'_2)^2}$$

where r is the Euclidean distance between the points x and x$'$, x_1 and x_2 represent the coordinates of a location in space (e.g., longitude and latitude), and ℓ_s is the length scale (spatial one in this case). A large length scale parameter implies that function values at two points will remain highly correlated even when these points are relatively far apart in the input space, which typically results in smoother functions. The length scale can be prescribed, estimated, or computed based on independent data[2]. In our FBGP approach, we jointly estimate the spatial length scale and the $\boldsymbol{\lambda}$ parameter (i.e., scaling factors), enabling us to capture the dependencies between these parameters and fully characterize their uncertainty. We present the prior distribution for all the parameters in Text S2, including ℓ_s.

We use the squared exponential kernel for the temporal covariance to express the relationship between two temporal points:

$$k_{\text{temporal}}(\mathrm{x}, \mathrm{x}') = \exp\left(-\frac{(x_3 - x'_3)^2}{2\ell_t^2}\right)$$

where x_3 denotes the normalized time point, with the scale adjusted such that the onset of the observation period corresponds to 0 and its conclusion aligns with 1, and ℓ_t is the temporal length scale.

Text S2. Prior Distributions for Parameters and Likelihood Function

This section describes the prior distributions for the hyperparameters and the likelihood function used in the FBGP model. Figure S3 also presents the prior distributions for the parameters in a graphical format.

Scaling Factor

$$\boldsymbol{\lambda} \sim \text{TruncatedNormal}(\mu = \boldsymbol{\mu}_{\boldsymbol{\lambda}}, \sigma = \boldsymbol{\sigma}_{\boldsymbol{\lambda}})$$

where TruncatedNormal represents the truncated normal distribution, $\boldsymbol{\mu}_{\boldsymbol{\lambda}}$ is set to 1 for all four sectors, and $\boldsymbol{\sigma}_{\boldsymbol{\lambda}}$ is set to 0.3, 0.5, 0.5, and 0.3 for the FF, NEE, fire, and ocean sectors, respectively. The NEE and fire sectors, which are associated with more uncertainty, are assigned higher $\boldsymbol{\sigma}_{\boldsymbol{\lambda}}$ values.

Kernel Variance

$$\sigma^2 \sim \text{Gamma}(\alpha, \beta)$$

where α and β are parameters of a constrained Gamma distribution that draws random samples for σ^2 (see Equation 4 in the main text) between 0 and 0.1 ppm^2. Here, we set the upper bound for kernel variance at 0.1, which is lower than that of the noise parameter. This is due to the higher uncertainty assigned to the noise parameter, stemming from OCO measurement and transport errors. The reported OCO-3 retrieval error ranges from 0.23 to 2 ppm [3].

Spatial Kernel Length Scale

$$\ell_s \sim \text{Gamma}(\alpha, \beta)$$

where α and β are parameters for a constrained Gamma, which draws random samples for ℓ_s between 0 and d_{upper}. We set d_{upper} to 3 times the maximum distance between any spatial points within the domain.

Temporal Kernel Length Scale

$$\ell_t \sim \text{Exponential}(\lambda_{exp} = N_d/7)$$

where N_d is the total number of days in the year (in this case, the year 2020), and the number 7 refers to the typical synoptic weather cycle in days[4]. Note that the mean (or expected value) of an exponential distribution is given by the reciprocal of its rate parameter λ_{exp}.

Noise

$$\sigma_{noise} \sim \text{Exponential}\left(\text{scale} = \text{std}(\mathbf{y})\right)$$

where $\text{std}(\mathbf{y})$ represents the standard deviation of the target variable, and the scale parameter is used here, implying that the rate is the inverse of the standard deviation of **y**.

Likelihood

$$\mathbf{y} \sim \mathcal{N}(\boldsymbol{f}, \sigma_{\text{noise}}^2 \mathbf{I})$$

where $\boldsymbol{f}$ is the latent function, with its underlying function values treated as latent variables and a prior specified by the GP, and **I** is the identity matrix.

Text S3. **Scaling of Vulcan Fossil Fuel Emissions**

To accurately capture the fluctuations in emissions due to the pandemic lockdowns over various times of the year, specific adjustments were made to reflect the impact on sectors hit by the restrictions. For example, on-road emissions dropped sharply when lockdowns began, but by the end of 2020, they had rebounded to normal levels[5]. Scaling factors on a monthly basis were created for the on-road, aviation, and commercial marine vessel sectors based on actual usage data. The transportation sector utilized vehicle miles of travel (VMT) data from the CalTrans Performance Measurement System (PeMS, https://pems.dot.ca.gov), which was in agreement with previous findings that reductions in traffic due to the pandemic were consistent with declines in fuel sales[5]. Aviation activity was based on flight counts from the OpenSky network[6], and marine vessel activity used container throughput data from the California ports. These sector-specific scaling factors are presented in Table S1.

The 2015 Vulcan emissions by sector were converted to 2020 estimates by:

$$Vulcan_{2020}^{Month} = Vulcan_{2015}^{Month} \cdot R_{2020/2015}^{CARB} \cdot R^{Month}$$

where $Vulcan_{2020}^{Month}$ is estimated 2020 emissions, $Vulcan_{2015}^{Month}$ is the Vulcan emissions for 2015, $R_{2020/2015}^{CARB}$ is the ratio of CARB emissions for 2020 to 2015, and R^{Month} is the ratio of monthly activity to annual average activity by sector (see Table S2). For sectors other than onroad, aviation, and commercial marine vessels, the ratio is set to 1, adopting the base values.

Text S4. NEE, Fire and Ocean Prior Fluxes

We generated biospheric CO_2 fluxes, specifically the net ecosystem exchange (NEE), which represents the balance of CO_2 fluxes between ecosystem respiration (R_{eco}) and CO_2 uptake through gross primary productivity (GPP). We utilized estimates from the Solar-Induced Fluorescence for Modeling Urban biogenic Fluxes (SMUrF) model[7], which employs solar-induced fluorescence data (a proxy for GPP), CO_2 flux measurements from eddy covariance, and machine learning to estimate GPP and R_{eco}. The SMUrF model offers detailed hourly GPP and R_{eco} estimates.

We derived fire emissions from a global monthly product that estimates biomass burning and its associated fuel consumption and emissions[8]. This dataset utilizes data from the MODIS (MODerate resolution Imaging Spectroradiometer) Collection 6 satellite observations, following the Global Fire Emissions Database (GFED) framework. Initially available at a 500-meter resolution (https://zenodo.org/records/7229675), the dataset has been regridded to a coarser 2-km resolution to simplify data processing. Hourly fire emissions for each 2-km grid cell were calculated by integrating daily fractions and the diurnal cycle of fire emissions from GFED version 4.1 (https://daac.ornl.gov/VEGETATION/guides/fire_emissions_v4_R1.html), which provides data at three-hour intervals on a 0.25° grid.

We used ocean flux data from CarbonTracker version CT2022[9], with a 1° × 1° spatial resolution updated every three hours. Even though its impact within the state boundary is negligible, we adjusted the flux values to stay uniform throughout each three-hour segment.

All emission flux maps were further aggregated to a resolution of 0.5° × 0.625° for use as inputs in the GC CTM (see Section 2.3 of the main text and Text S5).

Text S5. GC for Source-Attributed CO_2 Simulations

The GC model was employed to simulate overall atmospheric CO_2 concentrations and source-specific contributions, including FF, NEE, fire, ocean, and boundary conditions, across the nested domain. Source-specific concentrations were determined by conducting sensitivity simulations, where individual source fluxes were turned off, and comparing the resulting CO_2 concentrations to the predictions from simulations that included all sources. Column-averaged CO_2 dry-air mole fractions (XCO2) simulated by the model for each OCO-2/3 retrieval (H) were obtained by convolving the model CO_2 profiles with the column-averaging kernel vector ($\boldsymbol{a}$) from OCO-2/3:

$$H = XCO2_a + \boldsymbol{a}^{\boldsymbol{T}}\boldsymbol{\phi}\big(\boldsymbol{\sigma}(\boldsymbol{x})\big) - \boldsymbol{c}_{\boldsymbol{a}}$$

where the prior profiles of CO_2 ($\boldsymbol{c}_{\boldsymbol{a}}$) and prior column CO_2 ($XCO2_a$) constitute prior information used in the OCO-2/3 XCO2 retrieval[10] and $\boldsymbol{\phi}\big(\boldsymbol{\sigma}(\boldsymbol{x})\big)$ refers to the GC-predicted vertical profiles of CO_2, interpolated to match the OCO-2/3 retrieval levels.

Text S6. **Marginal Likelihood for GP**

The marginal likelihood (before taking the logarithm) can be expressed as:

$$p(\mathbf{y}|\mathbf{x}) = \int p(\mathbf{y}|\boldsymbol{f}, \mathbf{x}) p(\boldsymbol{f}|\mathbf{x})\, d\boldsymbol{f}$$

where $p(\mathbf{y}|\mathbf{x})$ is the marginal likelihood of the observed data $\mathbf{y}$ given the inputs $\mathbf{x}$, $p(\mathbf{y}|\boldsymbol{f}, \mathbf{x})$ is the likelihood (not "marginal" because of the unknown function $\boldsymbol{f}$) of observing $\mathbf{y}$ given the function values $\boldsymbol{f}$ and inputs $\mathbf{x}$, $p(\boldsymbol{f}|\mathbf{x})$ is the prior (before seeing data) distribution of the function values specified by the GP model (see Figure 1 of the main text). The integral denotes the process of integrating out the function values $\boldsymbol{f}$, effectively summing over all possible configurations of $\boldsymbol{f}$ to focus on $\mathbf{y}$. In other words, $p(\mathbf{y}|\mathbf{x})$ is obtained by marginalizing over the latent functions $\boldsymbol{f}$. Thus, in our second approach (i.e., GP MLL), we adjust the hyperparameters of the GP model to maximize the log of the marginal likelihood, $p(\mathbf{y}|\mathbf{x})$, using GPyTorch (see Text S7 for implementation details).

From Equation 2 of the main text, the target variable $\mathbf{y}$ can be expressed as:

$$\mathbf{y} = \boldsymbol{f}(\mathbf{x}) + \epsilon$$

where $\epsilon \sim \mathcal{N}(0, \sigma_{\text{noise}}^2)$ and σ_{noise}^2 is the noise variance. Note that this noise accounts for measurement errors or other stochastic effects not captured by the deterministic part of the latent function $\boldsymbol{f}$ – i.e., random deviations from the deterministic part of the model.

The covariance function for $\boldsymbol{f}$ is given by:

$$\text{Cov}\big(f(\mathbf{x}), f(\mathbf{x}')\big) = k(\mathbf{x}, \mathbf{x}')$$

where $k(\mathbf{x}, \mathbf{x}')$ denotes the kernel function, which determines the covariance between the function values at distinct input points $\mathbf{x}$ and $\mathbf{x}'$. Consequently, for N training points, the resulting covariance matrix $\mathbf{K}$ forms an $N \times N$ matrix, where each element $\mathbf{K}_{ij} = k\big(\mathbf{x}_i, \mathbf{x}_j\big)$ represents the covariance between the function values corresponding to the ith and j^{th} input locations. This implies that we can sample from the prior distribution of $\boldsymbol{f}$ using the covariance matrix $\mathbf{K}$ before observing any data. Therefore, the prior distribution for $\boldsymbol{f}$ can be expressed as:

$$p(\boldsymbol{f}) = \mathcal{N}(\mathbf{0}, \mathbf{K})$$

where we assume a mean zero vector for simplicity. When we sample from $\mathcal{N}(\mathbf{0}, \mathbf{K})$, we generate a vector $\boldsymbol{f}$, where each element of $\boldsymbol{f}$ corresponds to the value of the function at the respective input $\mathbf{x}_i$. Thus, this vector $\boldsymbol{f}$ represents a specific, single realization of the stochastic process described by the GP model.

In GP modeling, no explicit assumptions are made about the form of the function $\boldsymbol{f}$. This contrasts with parametric methods that require predefined functional forms (e.g., linear, polynomial). As a nonparametric approach, GPs define a distribution over potential functions $\boldsymbol{f}$

(as shown in Figure 1), allowing for flexible data fitting without constraints on the function's type[11, 12].

Now, to obtain the marginal distribution of $\mathbf{y}$, which is Gaussian, we need expressions for $\mathbf{E}(\mathbf{y})$ and $\mathrm{Cov}(\mathbf{y})$. First, consider the expectation:

$$\mathbf{E}(\mathbf{y}) = \mathbf{E}(\boldsymbol{f}(\mathbf{x}) + \epsilon) = \mathbf{E}\big(\boldsymbol{f}(\mathbf{x})\big) + \mathbf{E}(\epsilon) = \mathbf{0}$$

where, as defined above, the mean of $\boldsymbol{f}$ is assumed to be zero for notational simplicity without limiting the generality, and the mean of ϵ is zero by definition.

The covariance of $\mathbf{y}$ for the marginal distribution can be expressed as:

$$\mathrm{Cov}(\mathbf{y}) = \mathrm{Cov}\big(\boldsymbol{f}(\mathbf{x})\big) + \mathrm{Cov}(\epsilon) = \mathbf{K} + \sigma_{\text{noise}}^2\mathbf{I}$$

where $\mathbf{K}$ represents the covariance matrix determined by the kernel function $k(\mathbf{x}, \mathbf{x}')$, and $\mathbf{I}$ is the identity matrix. The term $\sigma_{\text{noise}}^2\mathbf{I}$ accounts for the noise variance as described above.

Given the marginal distribution for the target $\mathbf{y}$,

$$p(\mathbf{y}|\mathbf{X}, \boldsymbol{\theta}) = \mathcal{N}(\mathbf{y}|\mathbf{0}, \mathbf{K}_\sigma)$$

where $\mathbf{K}_\sigma = \mathbf{K} + \sigma_{\text{noise}}^2\mathbf{I}$ and $\boldsymbol{\theta}$ includes other parameters influencing $\mathbf{K}$ (e.g., length scales). The probability density function for a multivariate normal distribution is given by:

$$p(\mathbf{y}|\mathbf{X}, \boldsymbol{\theta}) = \frac{1}{(2\pi)^{N/2}|\mathbf{K}_\sigma|^{1/2}} \exp\left(-\frac{1}{2}\mathbf{y}^T\mathbf{K}_\sigma^{-1}\mathbf{y}\right)$$

Taking the natural logarithm of the probability density function, we obtain the log likelihood:

$$\ell(\boldsymbol{\theta}) = \log p(\mathbf{y}|\mathbf{X}, \boldsymbol{\theta}) = -\frac{1}{2}\mathbf{y}^T\mathbf{K}_\sigma^{-1}\mathbf{y} - \frac{1}{2}\log|\mathbf{K}_\sigma| - \frac{N}{2}\log(2\pi)$$

where N is the number of data points in $\mathbf{y}$, $|\mathbf{K}_\sigma|$ is the determinant of the covariance matrix, and $\mathbf{K}_\sigma^{-1}$ is the inverse of the covariance matrix. This log likelihood function illustrates that the covariance matrix $\mathbf{K}_\sigma$, which includes contributions from both the kernel and noise, is dependent on the kernel parameters $\boldsymbol{\theta}$. This dependence highlights the role of $\boldsymbol{\theta}$ in shaping the overall structure and behavior of the GP model.

The marginal log likelihood is maximized (or minimize negative $\ell(\boldsymbol{\theta})$) to determine the optimal values of $\boldsymbol{\theta}$, thus refining the model parameters to achieve the best fit to the observed data. This parameter optimization is conducted using the built-in functionalities of GPyTorch.

Text S7. GP MLL Implementation

As outlined in the main text, we employed a traditional machine learning approach (i.e., training-validation approach) to optimize the hyperparameters of our GP model (i.e., for the GP MLL approach), implemented using GPyTorch. Specifically, we employed Optuna (https://optuna.org), a hyperparameter optimization framework, to fine-tune the hyperparameters of the GP kernels. Although these parameters can also be optimized using the MLL method (see Text S6), we opted for Optuna due to its systematic capabilities for exploring extensive hyperparameter spaces. This choice was motivated by Optuna's efficient and robust search algorithms, which parallel the underlying principles of MLL but enhance scalability and exploration.

To tune these covariance length scale hyperparameters, we adopted a training-validation approach within the Optuna framework, dividing the entire dataset into training and validation subsets, with 25% reserved for validation. Using the validation dataset enhances the robustness of our parameter estimates by preventing overfitting and ensures that our model remains generalizable across various data subsets.

Furthermore, the optimization of the mean function hyperparameter ($\boldsymbol{\lambda}$) and the noise parameter was conducted directly through GP MLL, utilizing the entire dataset rather than just the training subset. This approach was informed by our ability to set more accurate priors for $\boldsymbol{\lambda}$ and the noise based on our understanding of *a priori* emissions and observed values. Thus, we employed a two-step approach to optimizing the hyperparameters: first, the covariance length scales (using the Optuna approach), and second, the $\boldsymbol{\lambda}$ and noise parameters. Here, we have demonstrated one of many possible implementations. Readers may choose to estimate all hyperparameters exclusively using either Optuna, or a similar framework, or GP MLL.

Text S8. Uncertainty Estimation for GP MLL

The uncertainty estimates for the GP MLL method are computed using the Fisher Information Matrix (FIM) approach. While a detailed explanation of the FIM method is beyond the scope of this work, particularly as we expect the vast majority of readers are from the atmospheric science community, we provide an intuitive overview of our implementation. The FIM represents the negative expected value of the Hessian (∇^2) of the log-likelihood function [∇^2 log p(y|θ)] with respect to model parameters θ[13]:

$$\mathrm{FIM} = -E[\nabla^2 \log p(y|\theta)]$$

where p(y|θ) is the likelihood of observing our data *y* given parameters θ. Under asymptotic theory, the inverse of FIM approximates the covariance matrix of the parameter estimates: $\mathrm{Cov}(\theta) \approx \mathrm{FIM}^{-1}$, and the square root of its diagonal elements provides estimates of the parameter standard errors.

In our implementation, we utilize the automatic differentiation capabilities of GPyTorch to compute the FIM efficiently. Automatic differentiation allows us to calculate the exact derivatives of our model's output with respect to its parameters without manual derivation. This is particularly valuable for our application because computing the FIM requires calculating second derivatives of the loss function.

The uncertainty estimates are influenced by several key parameters in our implementation. We set a Gaussian prior on the parameters with a mean of 1.0, based on our prior belief about the emission inventory, and a prior strength of $1/0.3^2$, which corresponds to a standard deviation of 0.3 (the same as the uncertainty set for FF in our prior for the scaling factor). This prior helps regulate the uncertainty estimates and prevent them from becoming unreasonably large. A stronger prior will tend to reduce the estimated uncertainties and pull the estimates closer to the prior mean, whereas a weaker prior allows the data to have more influence on the uncertainty estimates.

Our results in Figure S4 suggest insights into the performance of the GP MLL uncertainty estimation approach. Using a prior with a mean of 1.0 and standard deviation of 0.3, we obtained standard errors that appear reasonable for the FF and NEE sectors at 68% confidence intervals, although they are larger than those of the FBGP method. However, we observed that the uncertainty estimates for fire emissions are notably optimistic compared to our FBGP estimates. This discrepancy is particularly important because the fully Bayesian (i.e., FBGP) approach suggests greater uncertainty in fire emissions, which is more realistic given the relatively small signal-to-noise ratio of fire emissions in our dataset. These findings suggest that while our FIM-based uncertainty estimation provides a computationally efficient approach, the results should be interpreted cautiously, particularly in sectors with weak signals. The FBGP method appears to provide more realistic uncertainty estimates in such cases by fully accounting for the posterior distribution of the parameters.

Text S9. Computational Costs for FBGP and GP MLL Across CPU and GPU Platforms

We compared execution times between FBGP implemented in PyMC and GP MLL implemented in GPyTorch. The experiment was repeated across 10 iterations on both CPU and GPU platforms. FBGP was configured with a single chain for the NUTS MCMC algorithm, utilizing 3000 tuning samples and 2000 actual samples for the data.

The results, illustrated in Figure S5, demonstrate substantial differences in computational efficiency between the two methods. On the CPU, FBGP exhibited an average execution time of 7.54 minutes, while GP MLL was completed in 0.94 minutes. Similarly, on the GPU, FBGP required 2.96 minutes compared to GP MLL's 0.66 minutes.

Two key findings emerge from our analysis. First, GP MLL consistently outperformed FBGP in terms of execution speed across both computing platforms. This performance difference is expected, given the greater computational complexity involved in FBGP's NUTS MCMC algorithm compared to GP MLL's more streamlined approach. Second, the benefits of GPU acceleration were more pronounced for FBGP, demonstrating a 61% reduction in execution time compared to its CPU implementation. In contrast, GP MLL showed a more modest improvement of 30% with GPU acceleration.

The current study focused on a dataset comprising 71 data points from California. Scaling this analysis to larger domains, such as the continental United States (e.g., 500 points), would result in a computation time theoretically scaling cubically ($O(N^3)$). For instance, expanding from 71 to 500 points would theoretically increase computation time by a factor of approximately 350 (i.e., $(500/71)^3$). However, this limitation can be partially mitigated through parallel computing strategies, which become particularly effective when implemented on modern GPU architectures. Also, our experiments utilized a T4 GPU on Google Colab, and the implementation of higher-performing GPUs would likely yield further improvements in computational efficiency (see Text 12 for hardware acceleration).

We note that this test result is specific to our FBGP and GP MLL models on Google Colab, and actual computational costs may differ depending on the model and the computational environment.

Text S10. Description of the Classical Bayesian Method

The CB (Classical Bayesian) method is modeled as a Bayesian linear model, utilizing the same notation for $\boldsymbol{y}$ and $\boldsymbol{K_X}$ as in our GP model:

$$\boldsymbol{y} = \boldsymbol{K_X}\boldsymbol{\lambda} + \boldsymbol{v}$$

where $\boldsymbol{y}$ represents the vector of the target variable (i.e., observations), $\boldsymbol{K_X}$ represents the prior model predictions, with one column for each sector, $\boldsymbol{\lambda}$ is the state vector (i.e., the scaling factors for adjusting prior fluxes) and $\boldsymbol{v}$ is a vector representing the model-data mismatch. This mismatch is characterized by a covariance matrix $\boldsymbol{R}$, i.e., $\boldsymbol{v} \sim N(\boldsymbol{0}, \boldsymbol{R})$ where N denotes the normal distribution. We model $\boldsymbol{R}$ as a diagonal matrix representing the total variance associated with all error sources.[14-17]

Under Gaussian assumptions, the posterior estimate for $\boldsymbol{\lambda}$ can be analytically determined:

$$\boldsymbol{\lambda}_{post} = \left(\boldsymbol{K}_{\boldsymbol{X}}^{T}\boldsymbol{R}^{-1}\boldsymbol{K}_{\boldsymbol{X}} + \boldsymbol{Q}_{\lambda}^{-1}\right)^{-1}\left(\boldsymbol{K}_{\boldsymbol{X}}^{T}\boldsymbol{R}^{-1}\boldsymbol{y} + \boldsymbol{Q}_{\lambda}^{-1}\boldsymbol{\lambda}_{prior}\right)$$

where $\boldsymbol{\lambda}_{prior}$ is the a priori estimate for $\boldsymbol{\lambda}$ (initially set to one for all elements), and $\boldsymbol{Q}_{\boldsymbol{\lambda}}$ is the error covariance matrix for $\boldsymbol{\lambda}$. The corresponding posterior covariance for $\boldsymbol{\lambda}$ is given by:

$$\boldsymbol{V}_{post} = \left(\boldsymbol{K}_{\boldsymbol{X}}^{T}\boldsymbol{R}^{-1}\boldsymbol{K}_{\boldsymbol{X}} + \boldsymbol{Q}_{\lambda}^{-1}\right)^{-1}$$

Text S11. Inversion Results Using Actual OCO-2/3 Observations

Figure S7 shows the inversion results using real OCO observations. The prior and posterior predictive checks demonstrate that when applied to real observations, the GP model produces results consistent with the observational data (Figures S7a and S7b). Specifically, the posterior predictive check confirms the model's ability to reproduce observations using the posterior hyperparameters.

Comparing predicted and observed CO_2 concentrations reveals a reduction in posterior error relative to the prior (see Figure S7c). This reduction suggests that the inversion process resulted in scaling factors that are more consistent with the observations than those derived from the prior.

The posterior scaling factors in Figure S7d indicate that while FF emissions remain consistent with the prior, the actual NEE drawdown (negative fluxes) is smaller than the prior estimate. As expected, both fire and ocean emissions exhibit large uncertainties due to their small signal-to-noise ratios.

Text S12. Hardware Acceleration and Performance Scaling for Future Atmospheric Inversions

In this work, we focused on demonstrating the effectiveness of GPU-JAX-based inference for atmospheric inverse modeling. Our current implementation already achieves significant computational gains through GPU acceleration and automatic differentiation, making it practical for operational use. We acknowledge, however, that emerging technologies such as quantum computing, advanced tensor processing units (TPUs), and distributed computing frameworks could further enhance performance.

As an example of the potential for better hardware technology, we conducted additional inversions using the more recent NVIDIA A100 GPU, which features 40 gigabytes (GB) of memory and was released in 2020 (https://www.nvidia.com/en-us/data-center/a100/). Note that our original analysis utilized NVIDIA's T4 GPU (https://www.nvidia.com/en-us/data-center/tesla-t4/), released in 2018 with a smaller memory capacity of 15 GB. The comparison, shown in Figure S8, indicates that the A100 provides approximately twice the performance speed of the T4. In future work specifically focused on computational optimization for atmospheric inverse problems, we will consider addressing these broader computational strategies and hardware improvements.

Text S13. CONUS-scale Covariance Structure for OCO-3 Observations

Our findings indicate that the GP framework could be extended to larger regions, such as the continental United States (CONUS). Figure S10 illustrates the covariance structure for OCO-3 observations in July 2023. To compute the covariance matrices, we used approximately 2000 independent OCO-3 observation points across space and time, as shown in Figure S9. For the spatial dimensions, we set the length scales to 300 km (~3°), and for the temporal dimension, we selected a length scale of 0.1 in normalized time units. These values were chosen to illustrate the covariance structure at the CONUS scale, although they need to be optimized in the actual inversions.

Figure S10a displays the normalized time coordinate for these observations, showing a data gap (i.e., missing) between normalized times of 0.25 to 0.5, with 0.5 representing the midpoint of the month. Figures S10b – S10d show that the combined spatiotemporal kernel (Figure S10d), which accounts for both temporal and spatial dimensions, differs significantly from those considering only one dimension. Specifically, the temporal kernel (Figure S10b) reveals a decay in correlation with increasing temporal distance between points, while the spatial covariance (Figure S10c) exhibits a periodic pattern that mirrors the satellite's repeated observations over the same locations. The spatiotemporal kernel (Figure S10d) results from the element-wise multiplication of the spatial and temporal kernels, though these kernels could also be combined in other ways, such as by addition or both addition and scaling[18].

Tables

Table S1. CARB-derived scaling factors for emissions in 2020 versus 2015 by sector.

Commercial	Electricity Production	Industrial	Nonroad	Onroad	Rail	Residential
0.93	0.79	0.85	1	0.84	0.73	1.11

Table S2. Monthly scaling factors (ratio to annual average) for selected fossil fuel sectors.

Month	Aviation	Commercial Marine Vessel	Onroad
1	1.55	1.00	1.14
2	1.52	0.77	1.09
3	1.29	0.70	0.95
4	0.51	0.86	0.74
5	0.60	0.85	0.89
6	0.77	0.91	0.98
7	0.95	1.11	1.04
8	0.99	1.16	1.05
9	0.91	1.16	1.03
10	0.96	1.22	1.08
11	0.97	1.13	1.00
12	0.99	1.15	1.00

Figures

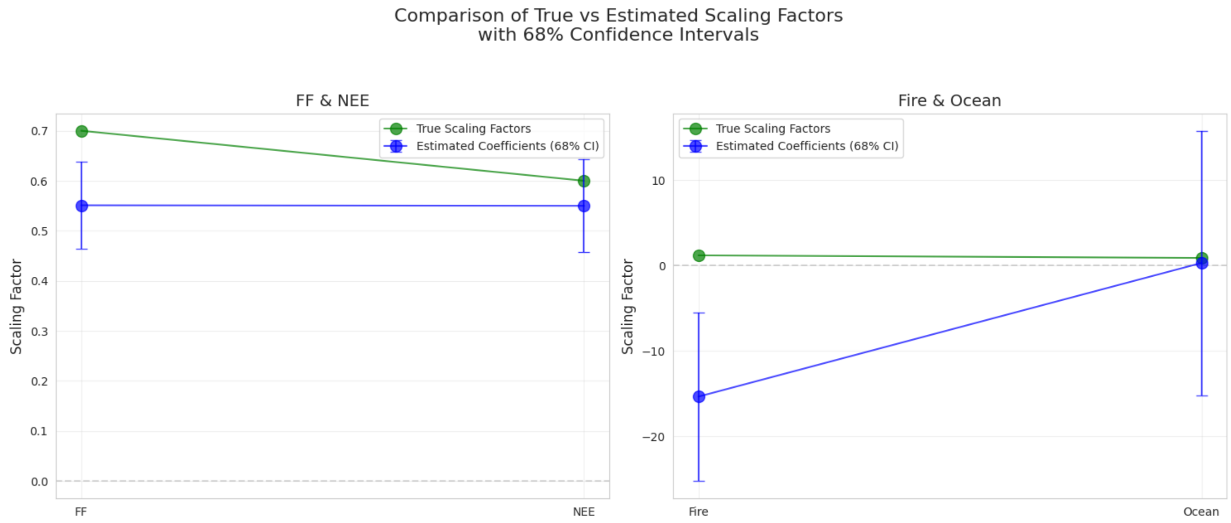

Figure S1. Results of a simple linear regression using the same data used in the GP inversions. Due to the scale difference, FF and NEE are shown separately from the fire and ocean sectors.

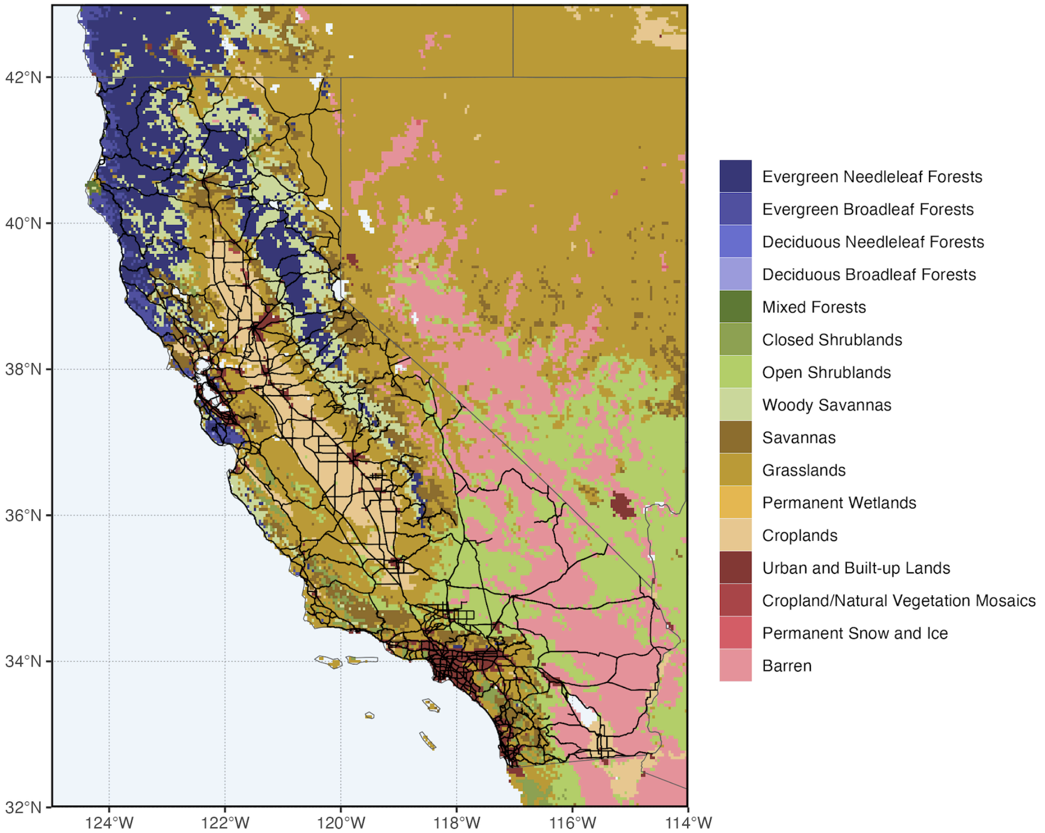

Figure S2. Land cover classification and major roads (black solid lines) for the year 2020. The land cover data comes from the MODIS (Moderate Resolution Imaging Spectroradiometer) MCD12Q1 product (version 006; https://lpdaac.usgs.gov/products/mcd12q1v006/). The figure displays California's primary and secondary roads, with data obtained from the U.S. Census Bureau (https://www.census.gov/cgi-bin/geo/shapefiles/index.php?year=2020&layergroup=Roads).

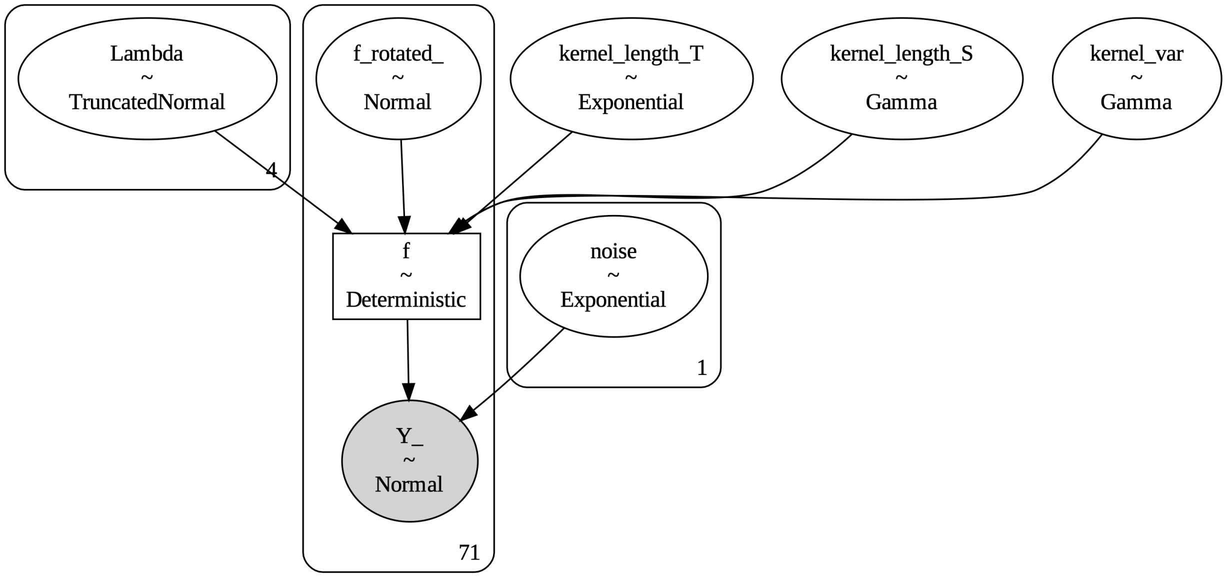

Figure S3. Graphical representation of the GP model for the FBGP method used in this study. The shaded area represents the observations, while the unshaded variables are modeled as latent variables to be estimated from the data. Details corresponding to this figure are presented in Text S2.

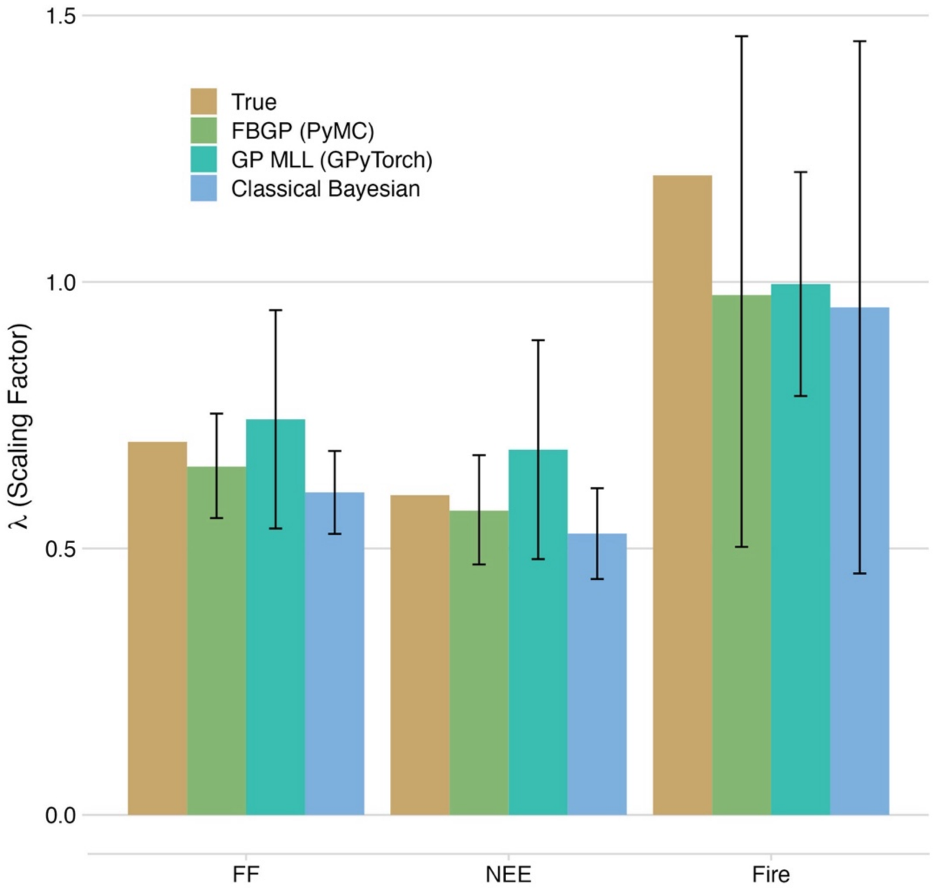

Figure S4. Comparison of true and inferred scaling factors by sector between FBGP, GP MLL, and CB. Error bars represent the 68% confidence intervals for all models.

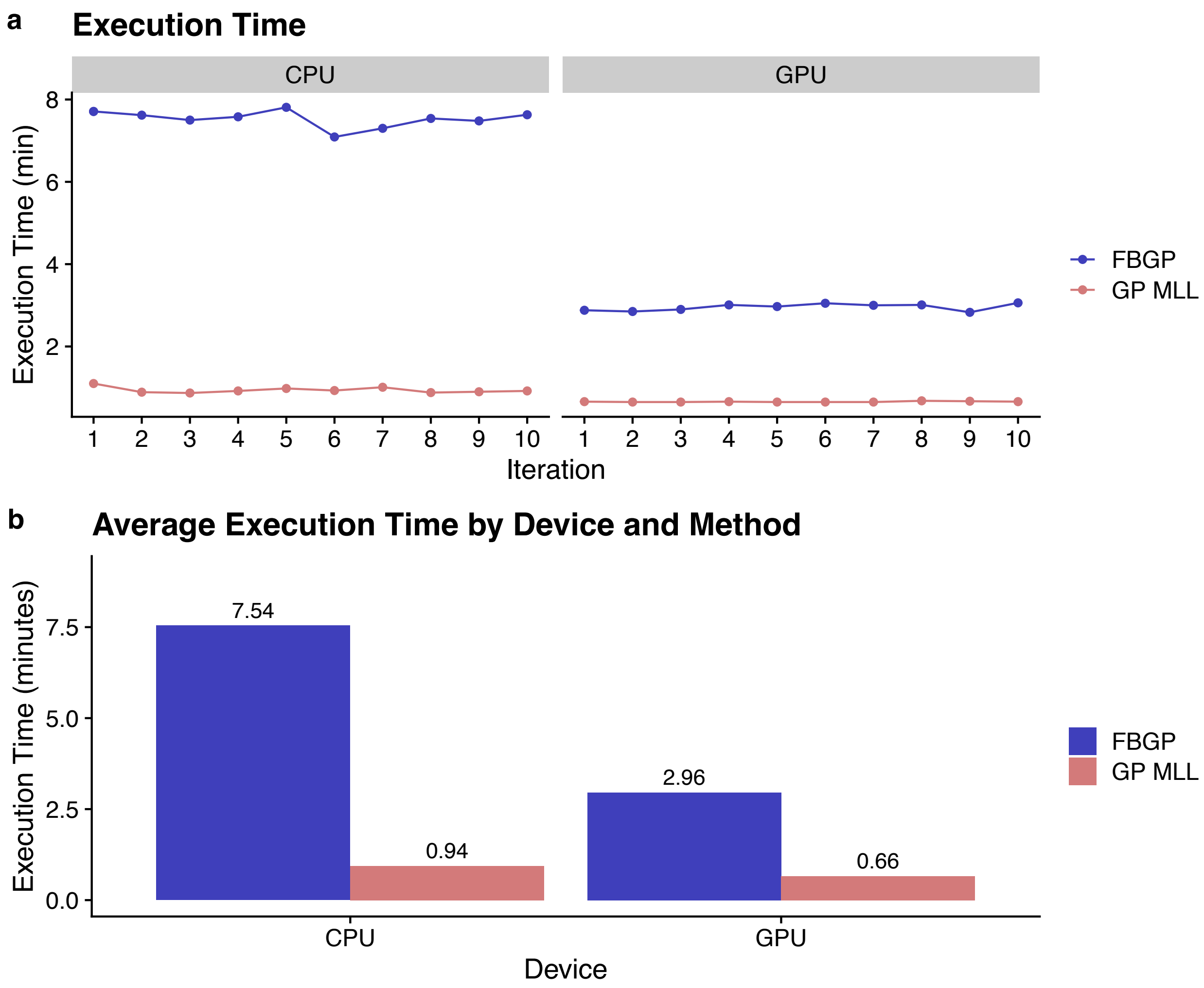

Figure S5. Comparison of computational costs between computing platforms (CPU vs. GPU) across GP methods: a) total execution time over 10 iterations, and b) average execution time per iteration.

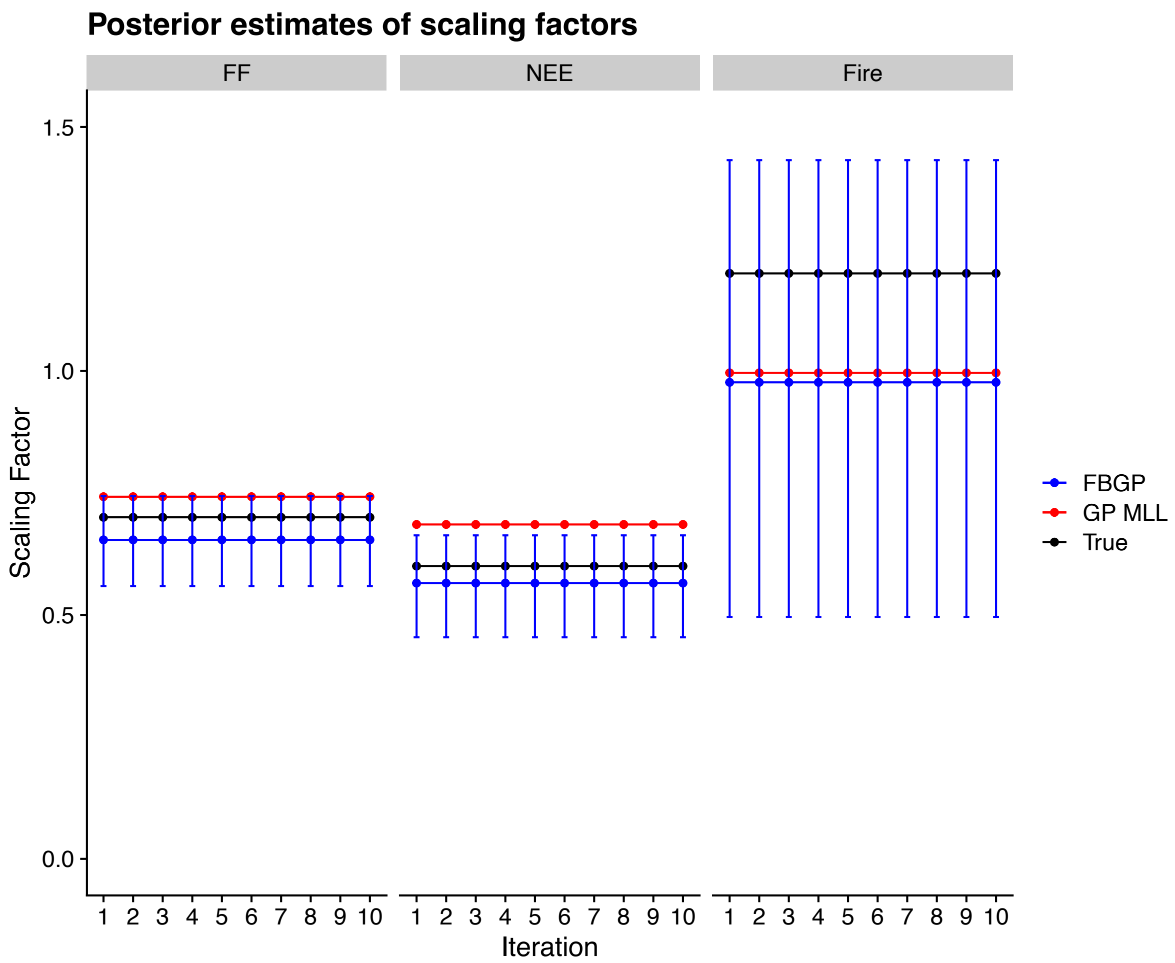

Figure S6. Convergence analysis of posterior scaling factors over 10 iterations for FF, NEE, and fire sectors. Results show consistent estimates across iterations for both FBGP (blue) and GP MLL (red) methods, compared to true values (black). For GP MLL, only point estimates are shown.

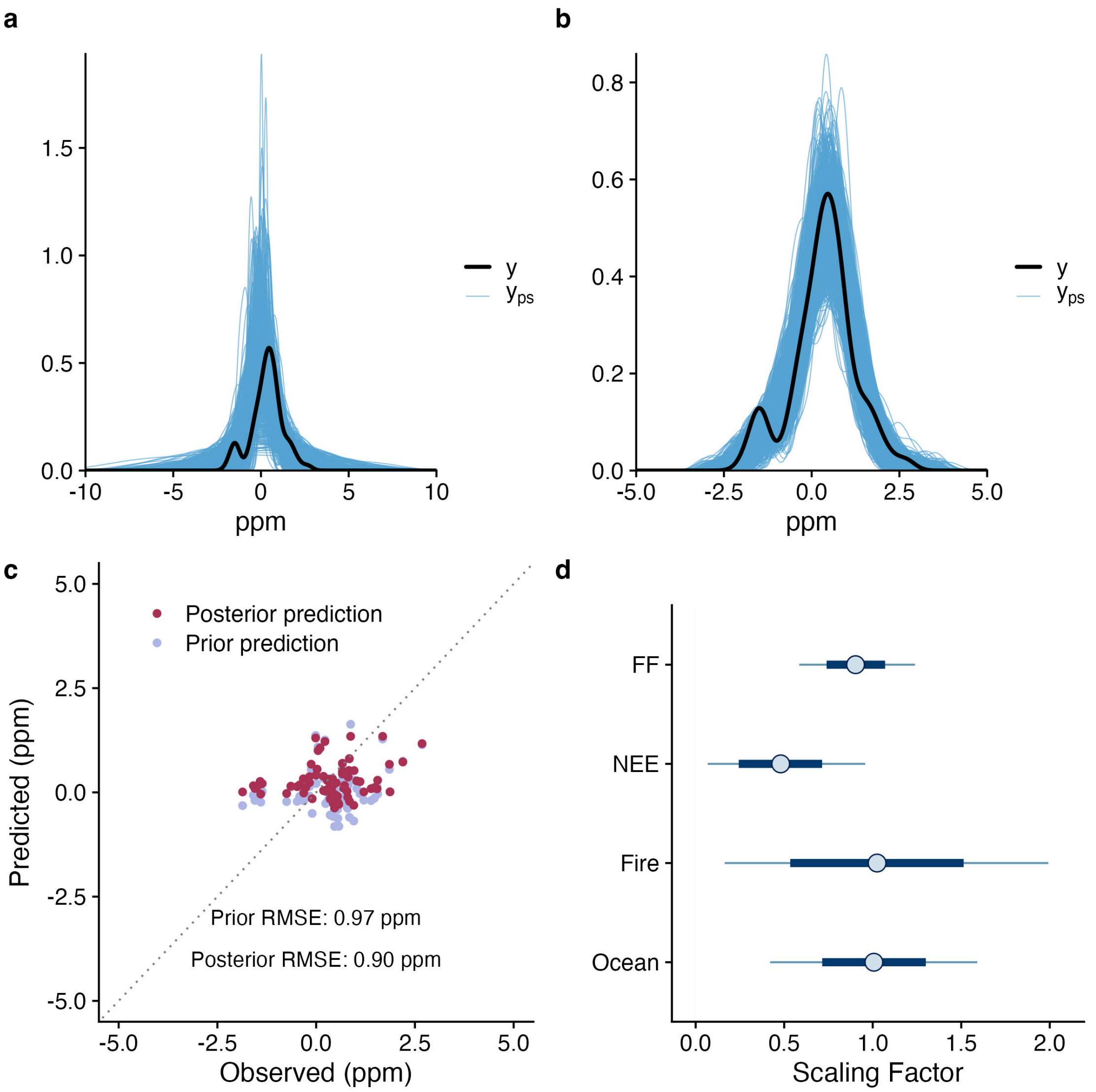

Figure S7. Inversion results using real OCO-2/3 observations from July 2020: a) prior predictive check, b) posterior predictive check, c) comparison of predicted and observed background-subtracted CO_2 concentrations and d) estimated posterior scaling factors. In a) and b), the thick solid line represents the observations. In d), the inner thick bars represent the 68% confidence interval, while the extended bars indicate the 95% confidence interval.

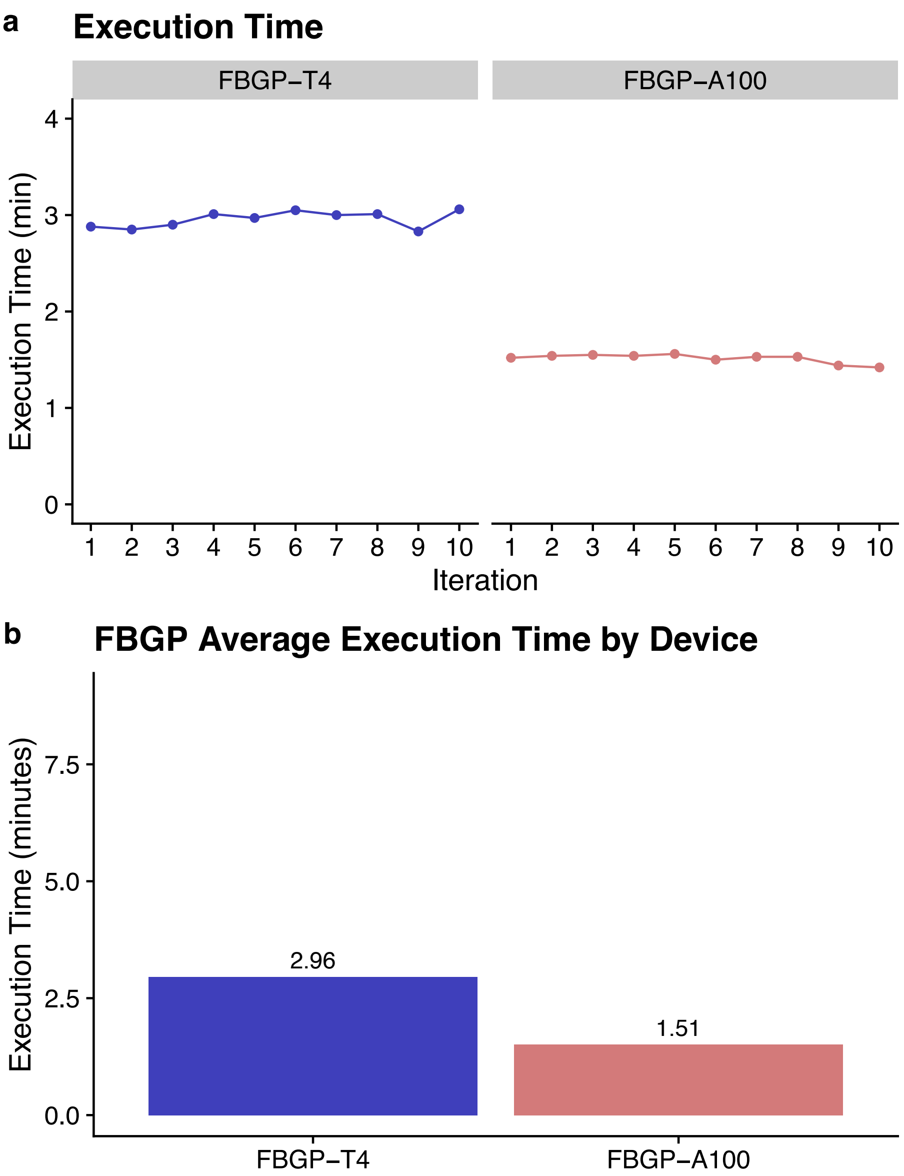

Figure S8. Comparison of FBGP inversion execution times between T4 and A100 GPUs: a) execution time across 10 inversions, and b) average execution time for FBGP inversions by GPU device.

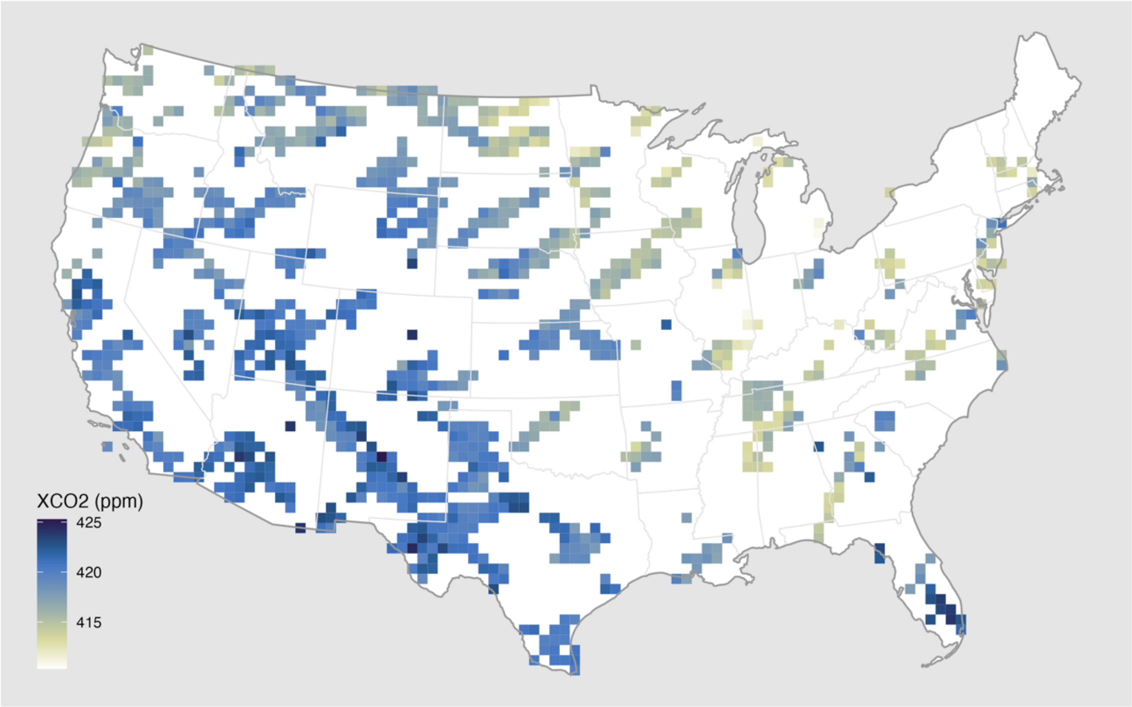

Figure S9. OCO-3 observations from July 2023 aggregated at a spatial resolution of 0.45°.

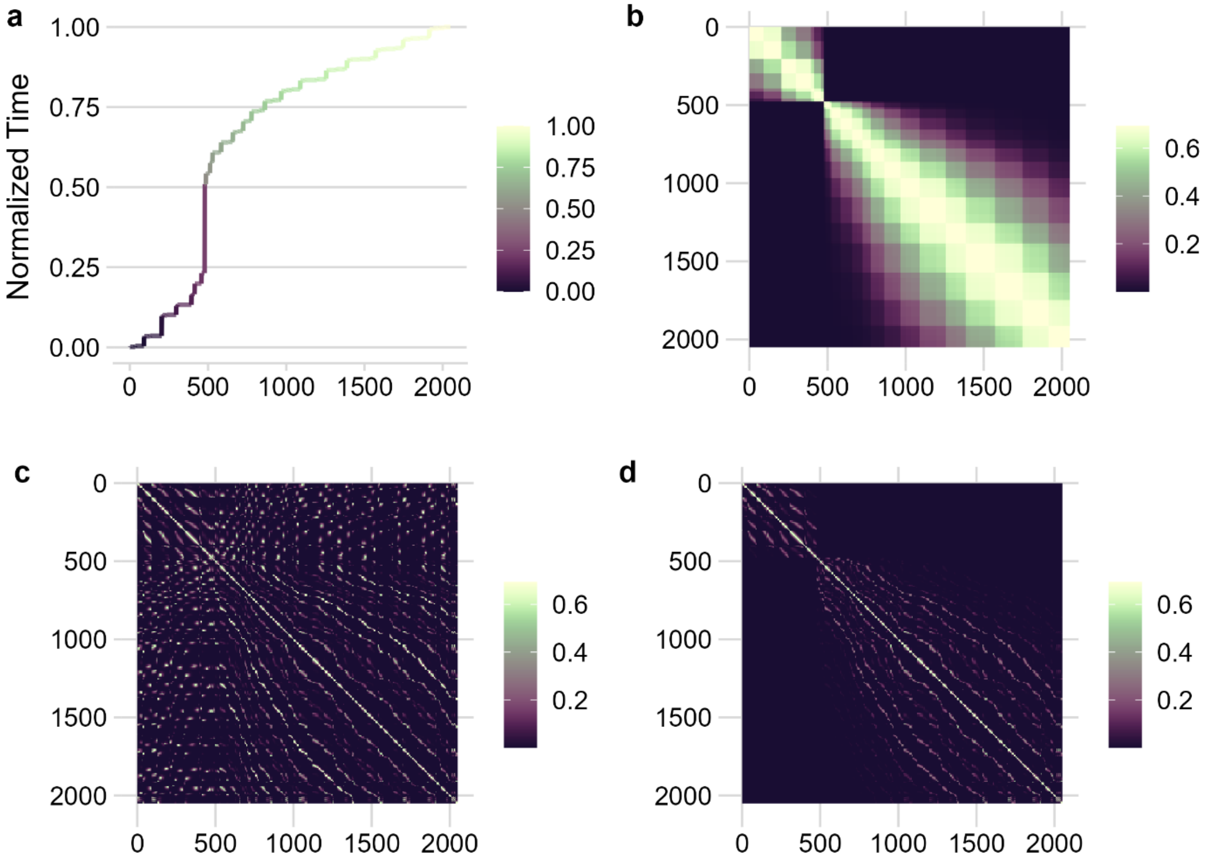

Figure S10. Temporal distance and kernels using OCO-3 observations over CONUS in July 2023: a) normalized time coordinate for July 2023, b) temporal kernel, c) spatial kernel, and d) spatiotemporal kernel. The temporal and spatial covariances were constructed using the squared exponential and Matérn 5/2 kernels, respectively. In this GP kernel evaluation, the kernel variance was not applied, making the kernel output dimensionless.

References

(1) Bevilacqua, M.; Caamaño-Carrillo, C.; Porcu, E. Unifying compactly supported and Matérn covariance functions in spatial statistics. *J. Multivar. Anal.* **2022**, *189*, 104949. DOI: https://doi.org/10.1016/j.jmva.2022.104949.
(2) Baker, D. F.; Bell, E.; Davis, K. J.; Campbell, J. F.; Lin, B.; Dobler, J. A new exponentially decaying error correlation model for assimilating OCO-2 column-average CO2 data using a length scale computed from airborne lidar measurements. *Geosci Model Dev* **2021**, *15* (2), 649-668. DOI: 10.5194/gmd-15-649-2022.
(3) Roten, D.; Lin, J. C.; Das, S.; Kort, E. A. Constraining Sector-Specific CO2 Fluxes Using Space-Based XCO2 Observations Over the Los Angeles Basin. *Geophysical Research Letters* **2023**, *50* (21). DOI: 10.1029/2023gl104376.
(4) Ganesan, A. L.; Rigby, M.; Zammit-Mangion, A.; Manning, A. J.; Prinn, R. G.; Fraser, P. J.; Harth, C. M.; Kim, K. R.; Krummel, P. B.; Li, S.; Mühle, J.; O'Doherty, S. J.; Park, S.; Salameh, P. K.; Steele, L. P.; Weiss, R. F. Characterization of uncertainties in atmospheric trace gas inversions using hierarchical Bayesian methods. *Atmospheric Chemistry and Physics* **2014**, *14* (8), 3855-3864. DOI: https://doi.org/10.5194/acp-14-3855-2014.
(5) Harkins, C.; McDonald, B. C.; Henze, D. K.; Wiedinmyer, C. A fuel-based method for updating mobile source emissions during the COVID-19 pandemic. *Environ Res Lett* **2021**, *16* (6), 065018. DOI: 10.1088/1748-9326/ac0660.
(6) Strohmeier, M.; Olive, X.; Lübbe, J.; Schäfer, M.; Lenders, V. Crowdsourced air traffic data from the OpenSky Network 2019–2020. *Earth System Science Data* **2021**, *13* (2), 357-366. DOI: 10.5194/essd-13-357-2021.
(7) Wu, D.; Lin, J. C.; Duarte, H. F.; Yadav, V.; Parazoo, N. C.; Oda, T.; Kort, E. A. A model for urban biogenic CO2 fluxes: Solar-Induced Fluorescence for Modeling Urban biogenic Fluxes (SMUrF v1). *Geosci Model Dev* **2021**, *14* (6), 3633-3661. DOI: 10.5194/gmd-14-3633-2021.
(8) Wees, D. v.; Werf, G. R. v. d.; Randerson, J. T.; Rogers, B. M.; Chen, Y.; Veraverbeke, S.; Giglio, L.; Morton, D. C. Global biomass burning fuel consumption and emissions at 500-m spatial resolution based on the Global Fire Emissions Database (GFED). *Geoscientific Model Dev Discuss* **2022**, *2022*, 1-46. DOI: 10.5194/gmd-2022-132.
(9) Jacobson, A. R.; Schuldt, K. N.; Tans, P.; Andrews, A.; Miller, J. B.; Oda, T.; Mund, J.; Weir, B.; Ott, L.; Aalto, T.; Abshire, J. B.; Aikin, K.; Aoki, S.; Apadula, F.; Arnold, S.; Baier, B.; Bartyzel, J.; Beyersdorf, A.; Biermann, T.; Zimnoch, M. CarbonTracker CT2022. **2023**.
(10) O'Dell, C. W.; Connor, B.; Bösch, H.; O'Brien, D.; Frankenberg, C.; Castano, R.; Christi, M.; Eldering, D.; Fisher, B.; Gunson, M.; McDuffie, J.; Miller, C. E.; Natraj, V.; Oyafuso, F.; Polonsky, I.; Smyth, M.; Taylor, T.; Toon, G. C.; Wennberg, P. O.; Wunch, D. The ACOS CO₂ retrieval algorithm – Part 1: Description and validation against synthetic observations. *Atmospheric Measurement Techniques* **2012**, *5* (1), 99-121. DOI: 10.5194/amt-5-99-2012.
(11) Wang, J. An Intuitive Tutorial to Gaussian Process Regression. *Computing in Science & Engineering* **2023**, *25* (4), 4-11. DOI: https://doi.org/10.1109/MCSE.2023.3342149.
(12) Rasmussen, C. E.; Williams, C. K. I. *Gaussian Processes for Machine Learning*; MIT Press, 2005.
(13) Murphy, K. P. *Probabilistic Machine Learning: An Introduction*; MIT Press, 2022.
(14) Gerbig, C.; Lin, J.; Wofsy, S.; Daube, B.; Andrews, A.; Stephens, B.; Bakwin, P.; Grainger, C. Toward constraining regional-scale fluxes of CO2 with atmospheric observations over a

continent: 2. Analysis of COBRA data using a receptor-oriented framework. *Journal of Geophysical Research: Atmospheres* **2003**, *108* (D24). DOI: https://doi.org/10.1029/2003JD003770.
(15) Zhao, C.; Andrews, A. E.; Bianco, L.; Eluszkiewicz, J.; Hirsch, A.; MacDonald, C.; Nehrkorn, T.; Fischer, M. L. Atmospheric inverse estimates of methane emissions from Central California. *Journal of Geophysical Research: Atmospheres* **2009**, *114* (D16).
(16) Göckede, M.; Michalak, A. M.; Vickers, D.; Turner, D. P.; Law, B. E. Atmospheric inverse modeling to constrain regional-scale CO2 budgets at high spatial and temporal resolution. *Journal of Geophysical Research: Atmospheres* **2010**, *115* (D15).
(17) Jeong, S.; Hsu, Y.-K.; Andrews, A. E.; Bianco, L.; Vaca, P.; Wilczak, J. M.; Fischer, M. L. A multitower measurement network estimate of California's methane emissions. *Journal of Geophysical Research: Atmospheres* **2013**, *118* (19), 11,339-311,351. DOI: https://doi.org/10.1002/jgrd.50854.
(18) Williams, C.; Rasmussen, C. Gaussian processes for regression. *Advances in neural information processing systems* **1995**, *8*.
